# Sensory deprivation in *Staphylococcus aureus*

**DOI:** 10.1038/s41467-018-02949-y

**Published:** 2018-02-06

**Authors:** Maite Villanueva, Begoña García, Jaione Valle, Beatriz Rapún, Igor Ruiz de los Mozos, Cristina Solano, Miguel Martí, José R. Penadés, Alejandro Toledo-Arana, Iñigo Lasa

**Affiliations:** 1 Instituto de Agrobiotecnología. Idab, UPNA-CSIC-Gobierno de Navarra, 31006 Pamplona, Spain; 2 Laboratory of Microbial Pathogenesis, Navarrabiomed, Universidad Pública de Navarra (UPNA), Complejo Hospitalario de Navarra (CHN), IdiSNA, Irunlarrea 3, 31008 Pamplona, Navarra Spain; 3 Instituto de Biomedicina de Valencia (IBV-CSIC), Jaume Roig 11, 46010 Valencia, Spain; 4 Institute of Infection, Immunity and Inflammation, University of Glasgow, Glasgow, G12 8TA UK; 5 Present Address: Department of Molecular Neuroscience, The Francis Crick Institute, University College London Institute of Neurology, WC1B 5EH, 1 Midland Road, London, NW1 1AT UK; 6 Present Address: Facultad de Veterinaria y Ciencias Experimentales, Universidad Católica de Valencia San Vicente Mártir, 46001 Valencia, Spain

## Abstract

Bacteria use two-component systems (TCSs) to sense and respond to environmental changes. The core genome of the major human pathogen *Staphylococcus aureus* encodes 16 TCSs, one of which (WalRK) is essential. Here we show that *S. aureus* can be deprived of its complete sensorial TCS network and still survive under growth arrest conditions similarly to wild-type bacteria. Under replicating conditions, however, the WalRK system is necessary and sufficient to maintain bacterial growth, indicating that sensing through TCSs is mostly dispensable for living under constant environmental conditions. Characterization of *S. aureus* derivatives containing individual TCSs reveals that each TCS appears to be autonomous and self-sufficient to sense and respond to specific environmental cues, although some level of cross-regulation between non-cognate sensor-response regulator pairs occurs in vivo. This organization, if confirmed in other bacterial species, may provide a general evolutionarily mechanism for flexible bacterial adaptation to life in new niches.

## Abstract (web summary)

Bacteria use two-component systems (TCSs) to sense and respond to environmental changes. Here, the authors show that *Staphylococcus aureus* can survive in the absence of all its 16 TCSs under growth arrest conditions, and each TCS seems to be sufficient to sense and respond to specific environmental clues.

## Introduction

A key factor that determines the evolutionary success of a living organism is the capacity to sense environmental factors and to respond accordingly. Thus, all organisms have evolved signal transduction mechanisms to establish “functional connectiveness” between environmental cues and cellular physiology. In the case of bacteria, two-component systems (TCSs) are the primary means of the sensorial machinery^1–3^. A canonical two-component signaling pathway contains a histidine kinase (HK), which in response to extracellular stimuli, autophosphorylates on a conserved histidine residue. The phosphorylated HK then binds and transfers the phosphoryl group to a conserved aspartate residue on the response regulator (RR). The phosphorylation of the RR activates an output domain, which can then effect changes in cellular physiology often by regulating gene expression, protein interactions, or enzymatic activities. The molecular mechanisms underlying TCS signal transduction processes have been exhaustively studied since the capacity of bacterial cells to sense and respond to changes in environmental conditions is critical to understanding bacterial biology and pathogenesis, and also because TCSs are considered suitable drug targets to treat bacterial infections^4^.

The genome of most clinically relevant bacterial species usually encodes multiple two-component HK-RR pairs, this number being proportional to the genome size, the diversity of environments in which organisms live, and the complexity in cellular differentiation^5,6^. Thus, bacteria inhabiting relatively stable host environments, such as obligate intracellular parasites, possess few or even none of these signaling systems, while bacteria able to live in a variety of environments and bacteria with complex lifestyles have dozens of unique TCSs, each one potentially responding to different stimuli and thus activating a different cellular response. The number of TCSs seems to expand primarily through a mechanism of gene duplication and subsequent accumulation of mutations that insulate the new pathways from the existing two-component pathways^7,8^. The final consequence of this evolutionary process is that bacteria gain the capacity to colonize a new niche or improve the efficiency to grow under the conditions of the existing niche. In principle, if the function of a TCS remains exclusively devoted to sensing and responding to a specific environmental signal, the process of acquiring TCSs should be reversible and the successive removal of TCSs should simply reduce the number of environmental conditions under which bacteria are able to grow. This assumption has never been tested because it is presumed that the accumulation of deleterious effects caused by successive mutation of TCSs would convert free-living bacteria into uncultivable species.

*Staphylococcus aureus* is an important human pathogen routinely isolated as a commensal organism living in different niches, including skin, nares, and mucosal surfaces of more than a third of the human population^9^. From these locations, *S. aureus* easily leads to infections ranging from relatively mild cutaneous infections to life-threatening infections such as pneumonia, sepsis, septic arthritis, endocarditis, and osteomyelitis in people predisposed with risk factors^10^. The versatility of *S. aureus* as a pathogen relies on its capacity to sense, respond, and adapt the expression of a large array of virulence factors in response to the environmental cues that bacteria encounter in each tissue^11–13^. For example, *S. aureus* is able to grow at a broad range of temperatures (from 4 to 44 °C), oxygen levels, pH (from 4.5 to 8), salt concentrations (up to 15% w/v NaCl), and desiccation conditions. Taking all the above into account, we selected *S. aureus* to investigate the following questions related to the TCS network: (i) which is the minimal number of TCSs needed to sustain life in a free-living bacteria?; (ii) are TCSs self-sufficient and autonomous entities?; and (iii) does TCSs cross talk occur in vivo?

Here, using a pioneering reductionist approach by which we removed all TCS genes in two genetically independent strains, we show that *S. aureus* deprived of the TCS network is able to survive under growth arrest conditions and that a single TCS is sufficient to sustain bacterial growth. We also show that each TCS works as a self-sufficient and autonomous module able to confer adaptation to a specific niche or environmental condition and that cross-regulation between non-cognate sensor-RR pairs, though rarely, occurs in vivo.

## Results

### Removal of the complete TCS network from *S. aureus*

**Fig. 1:**
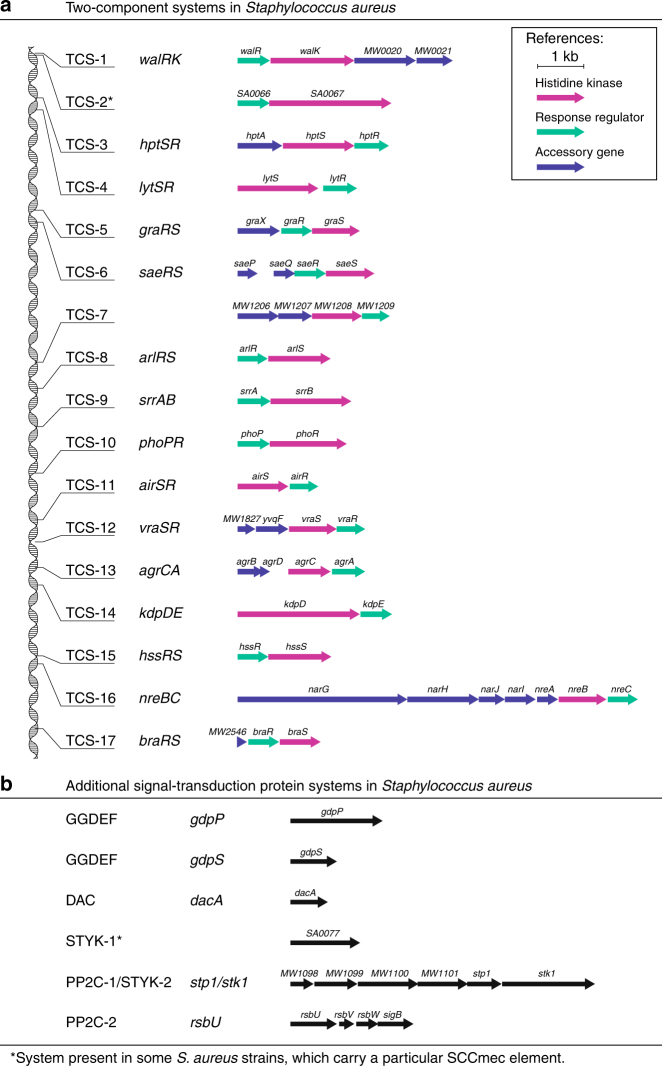
Signal transduction systems present in *S. aureus*. **a** Representation of the *S. aureus* genome with the 17 operons encoding two-component systems^14^ (http://www.ncbi.nlm.nih.gov/Complete_Genomes/SignalCensus.html). Histidine kinases are colored magenta, response regulators are colored green, and accessory genes are colored blue. Note that TCS-2 is only present in certain strains that carry a particular SCCmec element. **b** Additional signal transduction systems in *S. aureus*^14^ (http://www.ncbi.nlm.nih.gov/Complete_Genomes/SignalCensus.html). Genes and operons encoding the corresponding involved proteins in each signal transduction system are represented and colored black

**Fig. 2:**
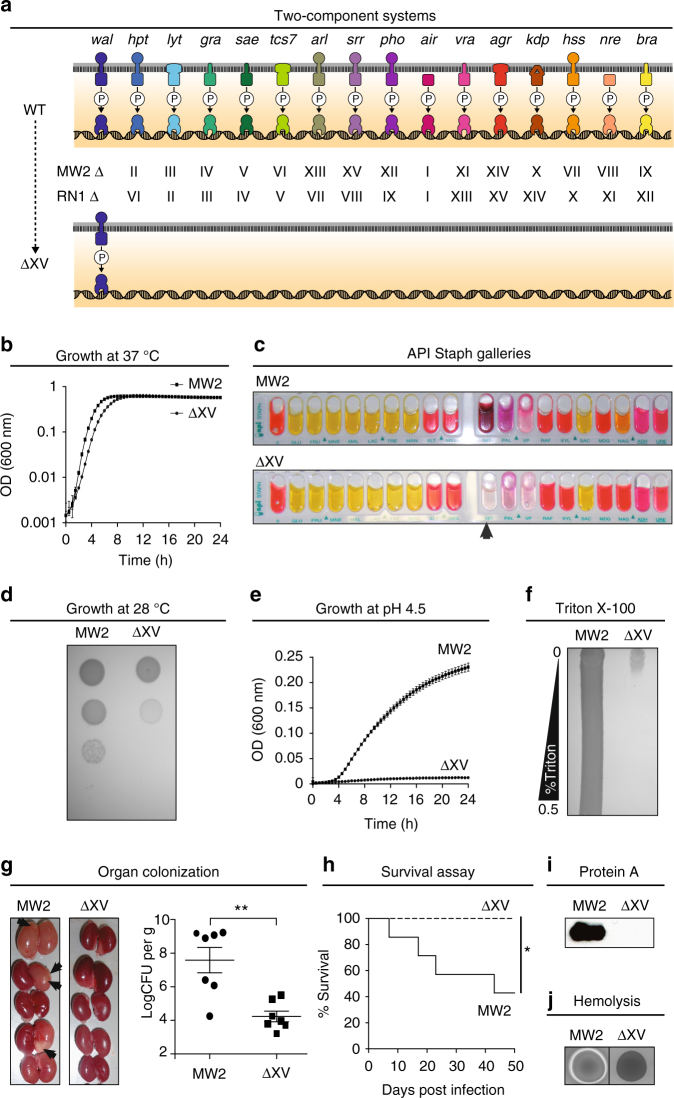
Phenotypic characterization of a *S. aureus* derivative only harboring the WalRK TCS. **a** Scheme of the TCSs present in wild-type (MW2 and RN1) and ΔXV strains. Latin numbers of intermediate mutants sequentially constructed in order to obtain ΔXV are indicated. **b** Growth curves in TSB medium at 37 °C. Average and SD of three independent assays are represented. MW2 doubling time: 34 min; ΔXV doubling time: 42 min. **c** Standard metabolic pathways analyzed using commercial API Staph galleries. Only the capacity to reduce nitrate to nitrite was affected in ΔXV strain (arrowhead). **d** Bacterial growth on TSA medium at 28 °C. Serial dilutions were spotted on agar plates. **e** Growth curves in TSB medium at pH 4.5. Average and SD of three independent assays are represented. **f** Triton X-100 sensitivity. Cultures were swabbed across agar plates containing a Triton X-100 concentration gradient. **g** Kidney colonization. Arrows indicate abscesses. Viable bacteria number per organ gram was determined. Average and SD are represented. Data were compared using the Mann–Whitney test (*n* = 7, **p < 0.01). **h** Survival of intravenously infected mice monitored for 7 weeks. Data were compared using a log-rank (Mantel–Cox) test (*n* = 7, *p < 0.05). **i** Protein A expression in chemically defined medium (CDM) detected by western blotting (full blot is shown in Supplementary Fig. 8). **j** Hemolysins production on sheep blood agar plates

We initiated this study in an attempt to confirm the essentiality of TCSs for bacterial survival by removing the complete TCS network from *S. aureus*. The core genome of *S. aureus* encodes 16 TCSs (http://mistdb.com, http://www.ncbi.nlm.nih.gov/Complete_Genomes/SignalCensus.html, http://www.p2cs.org)^6,14,15^ (Fig. 1), most of which are constitutively expressed under standard laboratory conditions (Supplementary Fig. 1). We sequentially deleted the 15 non-essential TCSs, including the HK- and the RR-coding genes, in two strain backgrounds, the methicillin-resistant *S. aureus* (MRSA) MW2 strain and the methicillin-sensitive *S. aureus* RN1 strain with a functional *rsbU* gene (Fig. 2a). Note that a different deletion order was used in each strain to avoid potential deleterious combinations. Deletion of the *walRK* genes was not attempted at the beginning of this study because it has been shown to be essential in *S. aureus*^16,17^. The genomes of the resulting strains, named as ΔXV, were sequenced to identify compensatory mutations that could have arisen during the successive deletion process. We identified 10 and 5 mutations in the MW2 and RN1 derivatives, respectively. However, the absence of common mutations between both strains indicated that the deletion process did not appear to select for particular genetic changes that influenced the behavior of the mutant strains (Supplementary Table 1). Remarkably, ΔXV mutant strains exhibited growth rates only slightly slower than that of the wild-type strains under aerobic conditions at 37 °C (Fig. 2b and Supplementary Fig. 2a). These data demonstrate that *S. aureus* harboring a single TCS, *walRK*, is viable and shows a very similar growth capacity compared to wild-type bacteria when grown under standard laboratory conditions, providing experimental evidence that most TCSs are dedicated to enhance the flexibility and adaptability of bacteria to environmental signals, not being involved in the control of essential cellular processes.

### Phenotypic characterization of strains deficient in TCSs

We next searched for phenotypes associated with the deletion of the TCS network in *S. aureus* ΔXV. Analysis of the metabolic capacities using API test revealed that *S. aureus* ΔXV strains exhibited a deficiency only in the capacity to reduce nitrate to nitrite (Fig. 2c and Supplementary Fig. 2b). In agreement with these results, a global metabolomic profile showed that the removal of the TCS network caused significant differences in the concentration of only a few metabolites (Supplementary Data 1). These metabolites are, in general, part of the metabolism of amino acids and carbohydrates but they do not fit into any specific metabolic pathway. *S. aureus* ΔXV also showed a growth defect at 28 °C (Fig. 2d and Supplementary Fig. 2c); a growth defect at pH 4.5 (Fig. 2e and Supplementary Fig. 2d); higher susceptibility to detergents (Triton X-100 gradient; Fig. 2f and Supplementary Fig. 2e); higher susceptibility to certain antibiotics (Supplementary Table 2); and a strong deficiency in the capacity to form abscesses in a mouse renal model (>1000-fold reduction in bacterial load, *p* < 0.01, Mann–Whitney test; Fig. 2g). Furthermore, *S. aureus* ΔXV strain was highly attenuated since all mice infected with the MW2 mutant strain survived after 50 days of infection, while 50% of the animals inoculated with MW2 wild-type strain died by day 21 post infection (Fig. 2h). Accordingly, the expression of virulence factors (protein A and hemolysins) was significantly reduced in ΔXV strains (Fig. 2i, j and Supplementary Fig. 2f, g). Taken together, these results confirmed that TCS signaling systems allow free-living bacteria to adapt to different environmental conditions, including life in animal hosts.

### TCSs are self-sufficient regulatory entities

**Fig. 3:**
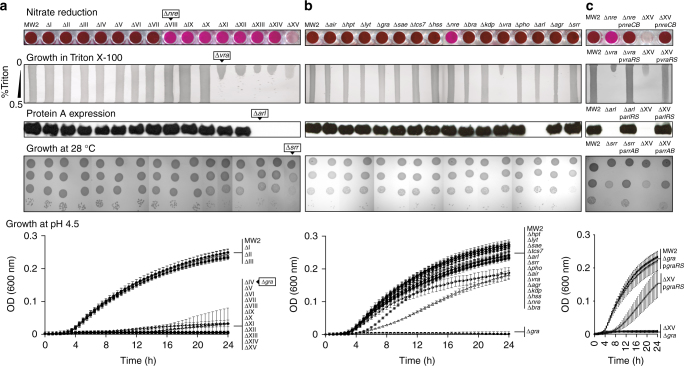
Genome-wide evaluation of modularity and self-sufficiency of TCSs in *S. aureus*. **a** Phenotypes of sequential TCS mutants: (i) nitrate reduction capacity, nitrite presence was colorimetrically detected; (ii) growth capacity on Triton X-100 concentration gradient agar plates; (iii) protein A expression in CDM detected by western blotting (full blots are shown in Supplementary Fig. 9); (iv) growth ability on TSA medium at 28 °C, serial dilutions were spotted on agar plates; (v) growth curves in TSB medium at pH 4.5, average and SD of three independent assays were recorded. TCSs whose mutation induces a phenotypic change are indicated. **b** Phenotypes of individual TCS mutants. Experimental conditions are described in **a**. **c** Phenotypes of selected individual and ΔXV mutant strains complemented with the corresponding TCS. Experimental conditions are described in **a**

**Fig. 4:**
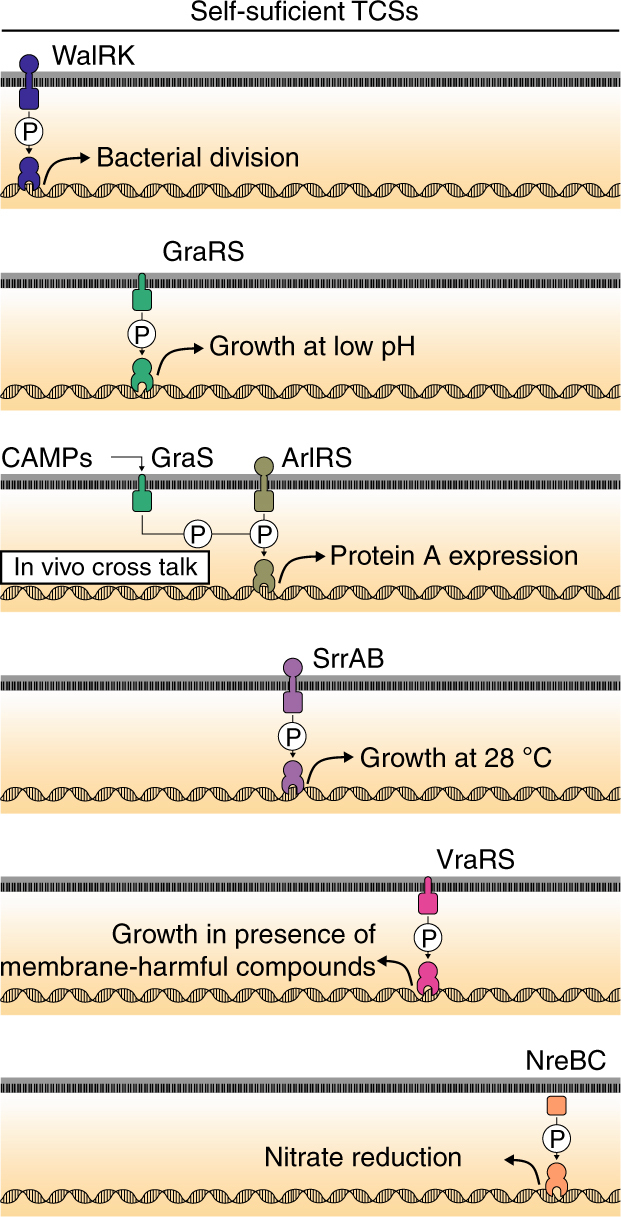
Schematic summary of the *S. aureus* strains producing a single TCS to demonstrate functional self-sufficiency. WalRK regulates bacterial division, GraRS is necessary to grow at a low pH, ArlRS induces the expression of protein A in CDM, SrrAB allows adaptation to grow at 28 °C, VraRS controls the susceptibility to Triton X-100, and NreBC confers the capacity to reduce nitrate to nitrite. Moreover, GraS is able to phosphorylate ArlR, upon the presence of antimicrobial cationic peptides, such as colistin, and activates protein A expression

To investigate how the complete TCS network operates as regards complementarity between different TCSs, we analyzed the aforementioned phenotypes both in a collection of single mutants in each TCS and in the collection of sequential mutants obtained during ΔXV construction (Fig. 3a, b). Surprisingly, we observed that the different ΔXV phenotypes appeared to be exclusively controlled by specific TCSs. Thus, the results revealed that NreBC confers the capacity to reduce nitrate to nitrite, VraSR is responsible for cell wall resistance to Triton X-100, SrrAB allows adaptation to grow at 28 °C, GraRS is necessary to grow at a low pH, and ArlRS is necessary for transcriptional activation of protein A expression in chemically defined media (CDM^18^ and RPMI). Note that protein A expression dependency on ArlRS in both chemically defined media and in three genetically unrelated *S. aureus* strains is the opposite to what it has been previously described in rich (trypticase soy broth; TSB) medium^19^ (Supplementary Fig. 3a and Supplementary Fig. 4a). We further explored the functional autonomy of TCSs by investigating the role of each TCS in the absence of other members of the network. For that, we made use of the ΔXV genetic background, which allows the generation of derivatives, each one exclusively containing the query TCS besides WalRK. Interestingly, the ectopic expression of a copy of the corresponding TCS was sufficient to fully rescue each phenotype in ΔXV strain (Figs. 3c and 4). Thus, NreCB restored the capacity to reduce nitrate to nitrite, VraRS restored the resistance to Triton X-100, SrrAB restored the capacity to grow at 28 °C, GraRS restored growth at a low pH, and ArlRS restored protein A expression. For the analysis of the phenotype associated to *S. aureus* ΔXV virulence deficiency, ectopic complementation was not a useful strategy because the presence of the plasmid is not guaranteed in the absence of antibiotic selective pressure during the infection process. Thus, the virulence phenotype was analyzed with selected derivatives of *S. aureus* ΔXV containing the *agr* system (ΔXV+*agr*) or the *srr* system (ΔXV+*srr*) restored in the chromosome. The *agr* system was chosen because of its major role in the production of proteases, hemolysins, and other virulence factors^20^, whereas the *srr* system was selected from its role in the adaptation to hypoxic and nitrosative stress encountered within the infected host^21^ (Supplementary Fig. 5). Evaluation of the capacity of these strains to form abscesses in a mouse renal model revealed that neither *agr* nor *srr* systems alone were capable to restore *S. aureus* virulence, despite the fact that the *agr* system rescued the production of hemolysins (probably δ-hemolysin, since *S. aureus* MW2 is a weak producer of α-hemolysin^22^). These results support the idea that survival in host tissues during infection very likely implies adaptation to more than one environmental condition and the production of several virulence factors, which consequently involves the participation of more than one TCS. Analysis of the dependency of specific virulence factors’ expression on particular TCSs requires further investigation.

Together, these results indicate that each TCS constitutes a self-sufficient regulatory entity capable of sensing, responding, and adapting to changes in a specific environmental condition. This functional autonomy also helps to explain why the phenotypic behavior of ΔXV strains is summarized as the sum of the individual phenotypes that depend on each TCS, without any synergistic cost to the bacterial fitness.

### Genome-wide cross-regulation among TCSs in vivo

**Fig. 5:**
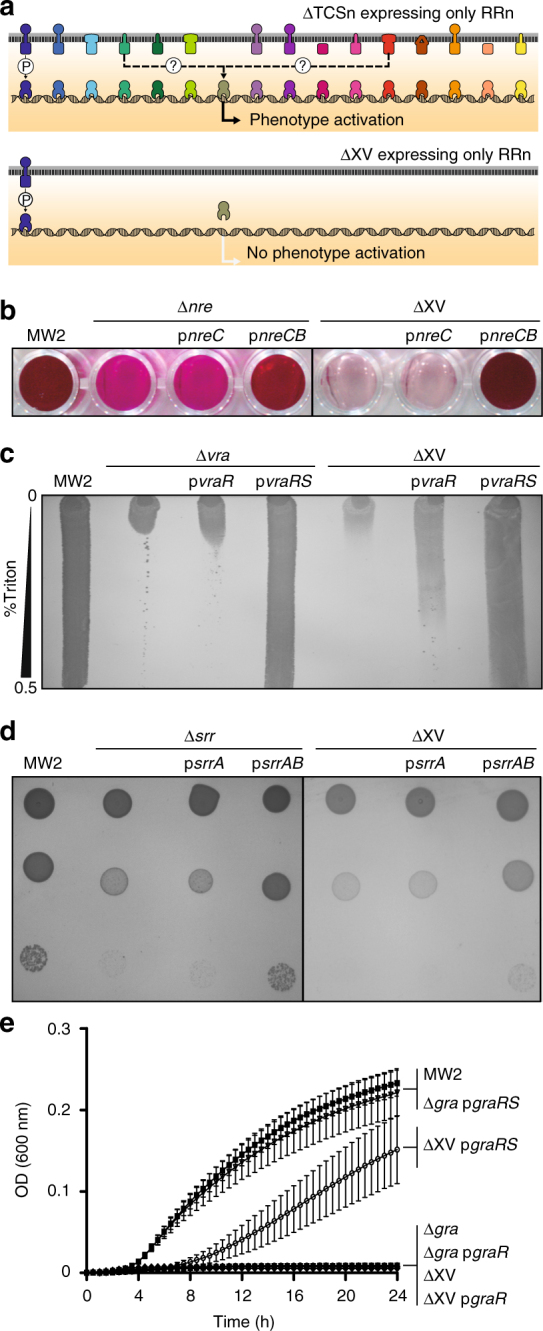
Analysis of TCS cross-activation in vivo. **a** Scheme of the followed strategy. Ectopic expression of a RR in the corresponding single TCS mutant and in ΔXV strain. A phenotype rescue in the single TCS mutant, but not in ΔXV strain, might indicate the cross-activation from non-cognate sensor kinases. This strategy was used to analyze the cross-activation of the corresponding phenotypes. **b** Nitrate reduction capacity. Nitrite presence was colorimetrically detected. **c** Growth capacity on Triton X-100 concentration gradient agar plates. **d** Bacterial growth on TSA medium at 28 °C. Serial dilutions were spotted on agar plates. **e** Growth curves in TSB medium at pH 4.5. Average and SD of three independent assays were recorded. Complementation with the complete TCS was used as a control in all sections

**Fig. 6:**
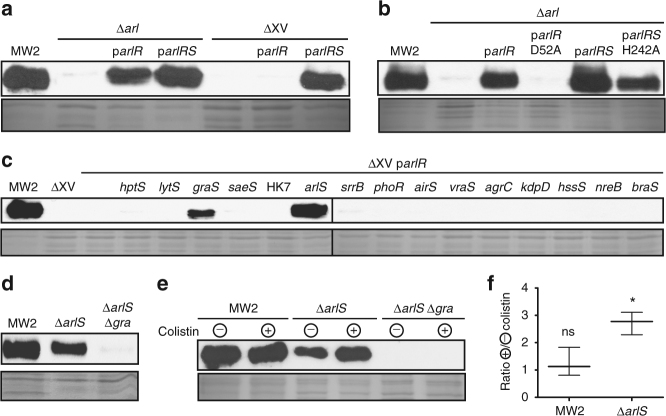
In vivo cross-talk analysis. Protein A expression in CDM was detected by western blotting on the following strains (full blots and gels are shown in Supplementary Fig. 10): **a** Δ*arl* and ΔXV strains, both complemented with *arlR* and *arlRS*; **b** Δ*arl* strain complemented with *arlR*, *arlR*(D52A), *arlRS*, and *arlRS*(H242A); **c** ΔXV strain complemented with the set of plasmids containing *arlR* combined with the 15 HKs; **d** MW2, Δ*arlS* and Δ*arlS* Δ*gra* strains; and **e** MW2, Δ*arlS* and Δ*arlS* Δ*gra* strains, in the presence or absence of colistin. It is known that colistin induces the activation of GraS. Coomassie-stained gels or Ponceau-stained membranes are shown as a loading control in all sections. **f** Densitometry quantification of the protein A western blotting bands. Ratios of protein A band intensity from MW2 and Δ*arlS* strains growing in the presence or absence of colistin were represented. Average and SD of three independent assays were recorded. Data were compared using two-tailed one-sample *t*-test inferred from a hypothetical value of 1 (*n* = 3, ns = no significant difference; *p < 0.05)

Studies on the specificity in the signal transduction phosphotransfer process have demonstrated that HKs typically exhibit a strong kinetic preference for their cognate RRs^23–29^. However, the extent to which cross-regulation occurs in vivo remains unclear, mainly because the presence of dozens of TCSs in a bacterial cell makes the analysis very complex. To answer this question, we reasoned that if ectopic expression of a native RR was able to rescue a phenotype in the corresponding single TCS mutant (defective both in the HK and the RR pair), but not in ΔXV strain, this might reflect the existence of cross talk from one or various non-cognate sensor HKs present in the single mutant but absent in ΔXV strain (Fig. 5a). To test this hypothesis, single mutant strains in TCSs for which a phenotype was identified (*nreCB*, *vraRS*, *arlRS*, *srrAB*, and *graRS*), as well as the ΔXV strain, were complemented with plasmids expressing the corresponding RRs. Note that the existence of a phenotype in the absence of the TCS implies that the TCS is active under the experimental conditions used in the analysis. The results revealed that plasmids expressing NreC, VraR, SrrA, and GraR were unable to restore the characteristic phenotype either in the single or in ΔXV mutant strains, at least under the laboratory growth conditions tested (Fig. 5). By contrast, ArlR was able to restore protein A expression in the single *S. aureus* Δ*arlRS* mutant but not in ΔXV mutant strain, suggesting the existence of a cross talk between ArlR and a non-cognate HK (Fig. 6a). We decided to further analyze this process. We first confirmed that phosphorylation of ArlR is required to induce protein A expression. For that, the single *arlRS* mutant strain was complemented with a plasmid expressing the non-phosphorylable ArlR D52A protein. As shown in Fig. 6b, ArlR D52A allele was unable to activate protein A expression. Because the phosphatase activity of HKs is, in many cases, a key mechanism for eliminating the nonspecific phosphorylation of their cognate RR, we analyzed the cross-activation of ArlR expressed in a strain carrying the ArlS H242A allele. According to previous studies with EnvZ, a HK from the same family as ArlS, replacement of the conserved histidine-242 would remove the kinase activity of ArlS preserving some level of phosphatase activity on ArlR^30,31^. Interestingly, the resulting strain showed a level of protein A expression similar to that observed in the single *arlRS* mutant strain complemented with ArlR, suggesting that the predicted phosphatase activity of ArlS H242A was unable to suppress cross-regulation of ArlR by other HKs (Fig. 6b). Alternatively, another explanation for these results is that mutation of the conserved histidine-242 might affect ArlS phosphatase activity.

We next sought to identify the HKs responsible for the cross-phosphorylation of ArlR in vivo. To address this, a set of 15 different plasmids, each one containing the *arlR* gene in combination with one non-cognate HK, were generated and used to transform the ΔXV strain (Fig. 6c and Supplementary Fig. 6). This strategy allowed us to identify GraS, in addition to ArlS used as a control of the experiment, as the HK capable to activate ArlR (Fig. 4 and Fig. 6c). As expected, activation of protein A expression disappeared in the double *arlS graRS* mutant (Fig. 6d, Supplementary Fig. 3b, and Supplementary Fig. 4b). Because the antibiotic colistin is able to activate GraS^32^, we tested whether stimulation of GraS with 50 μg ml^−1^ of colistin was capable of activating ArlR-dependent protein A expression (Fig. 6e, f). The results confirmed the activation of protein A synthesis in Δ*arlS* and not in the wild-type and Δ*arlS* Δ*graRS* strains. These data provide an example of cross-regulation that occurs at physiological protein levels and obeys to a natural input stimulus to the HK in vivo that is undetectable in the presence of the cognate ArlS. This is important because previous examples of cross-phosphorylation between a non-partner HK and RR were blind to input stimulus to the HK^33–36^. Interestingly, phosphorylation of ArlR by GraS does not appear to be due to a close phylogenetic relationship between them because the reciprocal phosphorylation, i.e., phosphorylation of GraR by ArlS does not detectably occur. Detailed system-level analysis with purified proteins in vitro have shown that small number of residues in the protein–protein interaction interface are responsible for the strong kinetic preference of the HK for transferring or removing the phosphate from the cognate RR pair^8,23,25,27,37^. Sequence identity between GraS and ArlS is 29% and 30% when the HK domain or the whole proteins are considered, respectively. Both proteins belong to the HisKA family of HK and they have been involved in the regulation of bacterial autolysis^38^, but they do not share the key residues on the α1 helix responsible for specificity^27^ (Supplementary Fig. 7). Overall, these results indicate that cross talk among different TCSs occurs in vivo.

### TCSs are dispensable when bacterial growth is arrested

**Fig. 7:**
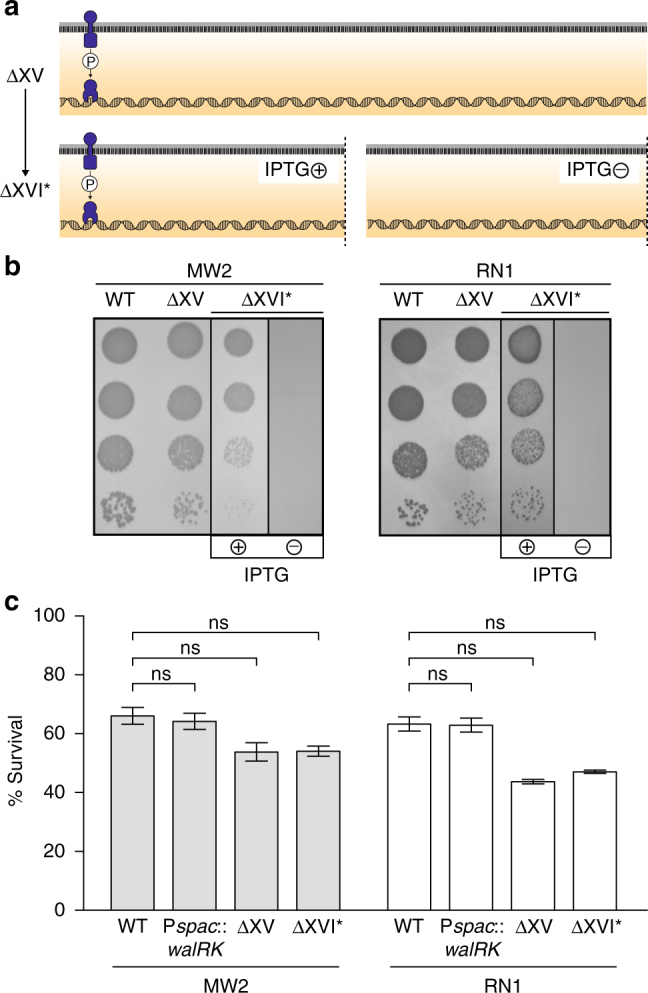
Consequences of the removal of the complete TCS network in *S. aureus*. **a** Scheme of the TCSs present in ΔXV and ΔXVI* strains. ΔXVI* strain harbors the *walRK* operon under the P*spac* IPTG-inducible promoter. IPTG presence in the media controls the expression of *walRK* in this strain. **b** Growth of *S. aureus* strains deficient in the TCS network at 37 °C in rich media. Drops of serial dilutions from bacterial cultures were spotted on TSA plates. IPTG was added to the media when indicated. **c** Survival of *S. aureus* ΔXVI* during starvation-induced growth arrest. The percentage of viable cells remaining after 7 days of growth arrest was determined. Average and SD of three independent assays were recorded. Data were compared using two-tailed nonparametric Mann–Whitney test (*n* = 3, ns = no significant difference)

Intrigued by the finding that the entire TCS network, with the exception of WalRK, can be removed without affecting *S. aureus* viability, we focused our studies on the WalRK system. For this, we replaced the promoter of the *walRK* genes with the isopropyl-β-d-thiogalactopyranoside (IPTG)-inducible P*spac* promoter in ΔXV strains, generating strains ΔXVI* (Fig. 7a). In the absence of IPTG, the expression of *walRK* is turned off and the cell is deprived of the complete sensorial TCS network. The ΔXVI* strains died when incubated in growth media without IPTG, confirming that *walRK* remains essential for bacterial viability in the absence of the remnant TCS network (Fig. 7b). For those bacteria living in multiple niches, it is very likely that they often end up in nutrient-scarce habitats. Nutrient deprivation causes growth arrest and profound physiological changes^39^. Thus, we sought to evaluate the necessity of WalRK TCS during starvation-induced growth arrest. Overnight cultures of *S. aureus* wild-type, ΔXV, and ΔXVI* strains (this last one grown in the presence of IPTG) were washed and resuspended in phosphate-buffered saline (PBS), dried on the surface of 24-well plastic plates, and incubated for 7 days at room temperature in the absence of IPTG. Bacterial viability was reduced to 65%, 55% and 55% for *S. aureus* wild-type, ΔXV, and ΔXVI* strains, respectively (Fig. 7c). Consistent with this, depletion of the WalRK system in the wild-type strain did not affect the capacity of bacteria to survive in these conditions. From these experiments, we concluded that the TCS signal transduction network is not required for coordinating all the changes necessary to adapt bacterial physiology to starvation and desiccation. Implicit in these results is that WalRK is essential only when bacteria are dividing but dispensable when bacterial growth is arrested. The existence of a TCS essential for bacterial viability is rare and somewhat counterintuitive in that it would seem to limit the conditions under which a bacterium can thrive. It can be speculated that some TCSs might assume the control of essential processes such as cell division, DNA replication, and autolysis, because this provides direct access to the machinery involved in stopping bacterial division when environmental conditions are not adequate for growth. In this regard, Delaune et al.^40^ showed that overexpression of a constitutively active form of WalR in *S. aureus* displayed a biphasic growth profile suggesting that constitutive WalR activation becomes detrimental once cells enter the stationary phase. Conversely, reduction of WalRK levels in the bacterial cell resulted in increased resistance to Triton X-100 and lysostaphin-induced cell lysis, and a significant decrease in peptidoglycan biosynthesis, turnover, and cell wall modifications^41^. It would be interesting to determine whether WalR plays a role in stopping bacterial division when environmental conditions are not appropriate for replication.

## Discussion

By using a systematic deletion approach, this work provides experimental evidence supporting the idea that free-living bacteria do not need to constantly monitor environmental and intracellular parameters for survival. In agreement with the fact that some obligate intracellular bacteria lack TCS signaling, our findings indicate that free-living bacteria can grow with a single TCS, even though growth in the laboratory still implies changes in culture parameters such as culture volume, dissolved oxygen concentration, nutrient and product concentrations, pH, and cell density. *S. aureus* ΔXV almost matches the definition of senseless bacterium. However, it still contains additional signal transduction systems (Fig. 1b) that include the following: (i) GdpS, the only GGDEF domain protein with a conserved GGDEF motif that is apparently unable to synthesize the secondary messenger c-di-GMP^42,43^; (ii) a c-di-AMP cyclase (Dac) and a phosphodiesterase (GpdP) involved in c-di-AMP metabolism^44^; and (iii) a Ser/Thr protein-kinase (Stk1) and two Ser/Thr protein-phosphatases (Stp1 and RsbU). Among these signal transducers, only Stk1 contains a transmembrane sensor domain that would be capable of responding to extracellular signals. Hence, though the capacity of *S. aureus* ΔXV strain to sense environmental cues is certainly highly restricted, it is not null.

Taking advantage of our genetic approach, we have analyzed genome-wide the specificity of TCS signaling pathways without the interference caused by other members of the network. Our data provide an example of cross-regulation of protein A expression that occurs at physiological protein levels and obeys to a natural input stimulus in vivo that is undetectable in the presence of the cognate sensor. In this case, and since protein A is a moonlighting protein playing different relevant roles during *S. aureus* infection^45^, the most likely interpretation is that different TCSs may converge in controlling the expression of a key gene, whose expression is absolutely required for bacteria under specific and different environmental conditions. A further step will require combining the systematic analysis of cross-regulation in vivo with the spatial localization of the regulatory sensor-regulator proteins.

We speculate that *S. aureus* ΔXV resembles a primitive staphylococcal ancestor that, only after successive and sequentially capturing TCSs, gained the capacity to colonize new environmental niches. The set of TCSs present in *S. aureus* is conserved in other closely related coagulase-negative staphylococcal species such as *Staphylococcus epidermidis* and *Staphylococcus haemolyticus*. However, *Staphylococcus saprophyticus*, a coagulase-negative staphylococcus that is a common inhabitant of the urinary tract, contains only 11 TCSs. *S. saprophyticus* frequently causes uncomplicated urinary tract infections in young and middle-aged female outpatients^46^ without the involvement of indwelling catheters. The fact that *S. saprophyticus* colonizes a very narrow niche of tissues compared to *S. aureus*, suggests that the different abilities of *S. aureus* and *S. saprophyticus* to colonize different body locations and cause infection may underlay at least partially in the number of TCSs. Empirical evidence that the acquisition of additional TCSs is a dynamic process is exemplified by the presence of a new TCS in some highly pathogenic *S. aureus* MRSA strains. The new TCS is homologous to the chromosomal copy of *kdpDE* and is carried in the SCCmec mobile element^47^. A definitive mechanistic insight in the process of acquisition of additional TCSs is likely to require long-term evolutionary experiments in which bacteria gain a selective advantage through the acquisition of a new TCS. We anticipate that the *S. aureus* ΔXV strain could represent an excellent model organism for this purpose.

## Methods

### Bacterial strains, plasmids, primers, and culture conditions

Bacterial strains, plasmids, and oligonucleotides are listed in Supplementary Table 3, Supplementary Table 4, and Supplementary Table 5, respectively. *Escherichia coli* strains were grown in LB broth (Pronadisa). *S. aureus* strains were grown in TSB (Pronadisa), the chemically defined medium CDM (Hussain-Hastings-White modified medium)^18^, and RPMI (Gibco Ref: 61870-010). When required for growth or selection, the medium was supplemented with IPTG, 1 mM, or/and appropriate antibiotics at the following concentrations: erythromycin, 1.5 and 10 µg ml^−1^; ampicillin, 100 µg ml^−1^; chloramphenicol 10 µg ml^−1^; and colistin, 50 µg ml^−1^.

### DNA manipulations and bacterial transformation

Routine DNA manipulations were performed using standard procedures unless otherwise indicated. Plasmids were purified using the NucleoSpin Plasmid miniprep kit (Macherey-Nagel) according to the manufacturer’s protocol. FastDigest restriction enzymes and Rapid DNA ligation kit (Thermo Scientific) were used according to the manufacturer’s instructions. Plasmids were transformed into *E. coli* XL1-Blue strain (Stratagene) by electroporation and then introduced in *S. aureus* by electroporation using a previously described protocol^48^. Staphyloccocal electrocompetent cells were generated as previously described^49^. Transformation of final *S. aureus* strains with pMAD and pSD3-3 plasmids were performed by phage transduction as described previously^50^. Note that for transductions of plasmids to MW2 and RN1 strains, ϕ85 phage and ϕ11 phage were used, respectively.

### Transcriptome analysis by tiling arrays

Complete transcriptome maps from several *S. aureus* strains obtained by tiling arrays and previously published^51^ (http://staph.unavarra.es/) were used to extract the transcriptional expression data from TCS operons. Images from IGB software showing the regions of the genome that encode the TCSs were extracted and shown separately (Supplementary Fig. 1).

### Allelic exchange of chromosomal genes

To generate deletion mutants, we amplified by PCR two fragments of at least 500 bp that flanked the left (primers A and B, Supplementary Table 5) and right sequences (primers C and D, Supplementary Table 5) of the region targeted for deletion. Chromosomal DNA from *S. aureus* 15981 strain was used as genomic template and Biotools DNA polymerase (Biotools) was used to carry out the PCR reactions. The PCR products (AB and CD fragments or AD fragments obtained by overlapping PCR, depending on the TCS, see Supplementary Table 5) were purified and cloned separately in the pGEM-T Easy vector (Promega). Cloned fragments were digested, purified, and fused by ligation into the shuttle vector pMAD^52^. pMAD ligations were electroporated into *E. coli* XL1-Blue (Stratagene) generating the pMAD::TCSsAD plasmid collection (Supplementary Table 4). Plasmids were purified from *E. coli* XL1-Blue and transformed into *S. aureus* RN4220 by electroporation and then introduced to MW2 and RN1 strains by phage transduction. Homologous recombination experiments were performed as described^53^. Erythromycin-sensitive white colonies, which did not further contain the pMAD plasmid, were tested by PCR using primers E and F (Supplementary Table 5).

The pMAD::TCS14AD(RN1) plasmid was constructed as described above, using kdp-RN1 primers (Supplementary Table 5) and RN1 chromosomal DNA as template. This plasmid was used to perform the allelic exchange of *kdpDE* genes in RN1 strain because the flanked sequences of the region targeted for deletion differed significantly in this strain.

To restore deleted TCSs in the chromosome of ΔXV strain, the TCS and the flanking sequences were amplified by PCR using primers A and D (Supplementary Table 5). Chromosomal DNA from *S. aureus* MW2 strain was used as genomic template and Phusion High-Fidelity DNA Polymerase (Thermo Fisher) was used to carry out the PCR reactions. The PCR products were purified and cloned in the pJET vector (Thermo Fisher Scientific). Cloned fragments were digested, purified, and fused by ligation into the shuttle vector pMAD^52^. pMAD ligations were electroporated into *E. coli* XL1-Blue (Stratagene) generating the pMAD::TCS plasmids (Supplementary Table 4). Plasmids were purified from *E. coli* XL1-Blue and transformed into *S. aureus* RN4220 by electroporation and then purified and introduced into MW2 ΔXV strain by electroporation. Homologous recombination experiments were performed as described^53^. Erythromycin-sensitive white colonies, which did not further contain the pMAD plasmid, were tested by PCR using primers E and F (Supplementary Table 5).

To generate the insertional mutation of the *walRK* operon, the mutation present in RN4220 P*spac-yycF* strain and generated by the insertion of the pSD3-3 plasmid^17^ was transferred by phage transduction to the wild-type and ΔXV strains, generating P*spac::walRK* and ΔXV P*spac::walRK* (ΔXVI*) strains in MW2 and RN1 strains. The resulting mutants harbored the entire *walRK* operon under the control of the P*spac* IPTG-inducible promoter and were grown in TSA supplemented with Clo (10 µg ml^−1^). IPTG 1 mM was added to allow the growth of these strains. Plasmid insertion was confirmed by PCR using pSD3.3.1/WalR-Fw primers (Supplementary Table 5).

### Identification of spontaneous mutations in ΔXV strains

Identification of genetic variations present in ΔXV strains relative to the corresponding wild-type strains was carried out by computational comparison of genomic sequences generated by high-throughput sequencing. Briefly, genomic DNA was prepared from an overnight culture of parental (MW2 and RN1) and mutant (MW2 ΔXV and RN1 ΔXV) strains grown in TSB at 37 °C, as described previously^54^ and sequenced on an Illumina Genome Analizer IIx instrument (Genomics Platform of CIBIR, La Rioja, Spain). Reads (6M–9M 150 bp) were assembled de novo using SOAPdenovo2 algorithm^55^. The reads were assembled into 466 contigs (>100 bp in size) corresponding to 2 864 441 bp in length, with a N50 value of 16 063 and a GC content of 32.67% for MW2; 236 contigs (>100 bp in size) corresponding to 2 856 644 bp in length, with a N50 value of 50 702 and a GC content of 32.76% for MW2 ΔXV; 283 contigs (>100 bp in size) corresponding to 2 839 577 bp in length, with a N50 value of 60 685 and a GC content of 32.80% for RN1; and 292 contigs (>100 bp in size) corresponding to 2 807 518 bp in length, with a N50 value of 79 329 and a GC content of 32.80% for RN1 ΔXV. Subsequently, contigs were ordered with Mauve^56^ and consensus genomic sequences containing 35 contigs for MW2, 12 contigs for MW2 ΔXV, 17 contigs for RN1, and 17 contigs for RN1 ΔXV were manually generated using SnapGene software version 3.0.3 (GSL Biotech LLC, Chicago, USA). Finally, spontaneous and generated mutations present in ΔXV strains relative to the corresponding wild-type reference sequences were analyzed using the computational pipeline *breseq*^57^. Spontaneous mutations generated during the construction of ΔXV strains are presented in Supplementary Table 1.

### Growth kinetics

To compare the growth kinetics of bacteria and their growth ability at a low pH, overnight cultures grown in TSB medium at 37 °C were diluted in TSB to an OD_600nm_ = 0.1, and 5 μl of the diluted culture were used to inoculate 96 microtiter wells (Thermo Fisher Scientific, Hemel Hempstead, UK) containing 195 μl of media per well (TSB pH 7 or pH 4.5, according to each case). Duplicates were inoculated for each strain tested. Plates were incubated at 37 °C for 24 h and the growth was monitored measuring the OD_600nm_ every 30 min using the thermostatizated spectrophotometer SpectraMAX 340PC (Molecular Devices). The average and SD of three independent assays were plotted.

### Biochemical characterization

API Staph galleries (BioMérieux) were used to analyze bacterial metabolic patterns, according to the manufacturer’s instructions. Homogeneous bacterial suspensions with turbidity equivalent to 0.5 McFarland were prepared using the API Staph Medium. The microtubes were then filled with the bacterial suspensions and the strip was incubated inside the incubation box at 37 °C for 24 h. Changes in bacterial metabolic patterns were analyzed comparing results obtained with corresponding mutants and with the wild-type *S. aureus* strain.

### Quantitative metabolomics

Hydrophilic interaction liquid chromatography (HILIC) was carried out on a Dionex UltiMate 3000 RSLC system (Thermo Fisher Scientific) using a ZIC-pHILIC column (150 mm × 4.6 mm, 5 μm column, Merck Sequant). The column was maintained at 30 °C and samples were eluted with a linear gradient (20 mM ammonium carbonate in water, A, and acetonitrile, B) over 26 min at a flow rate of 0.3 ml min^−1^ as follows: 0 min 20/80 (%A/%B); 15 min 80/20 (%A/%B); 15 min 95/5 (%A/%B); 17 min 95/5 (%A/%B); 17 min 20/80 (%A/%B); and 24 min 20/80 (%A/%B). The injection volume was 10 μl and samples were maintained at 5 °C prior to injection. For the mass spectrometry (MS) analysis, a Thermo Orbitrap QExactive (Thermo Fisher Scientific) was operated in polarity switching mode and the MS settings were as follows: resolution 70 000; AGC 1e6; *m*/*z* range 70–1050; sheath gas 40; auxiliary gas 5; sweep gas 1; probe temperature 150 °C; and capillary temperature 320 °C.

For positive mode ionization: source voltage +3.8 kV; S-Lens RF Level 30.00; S-Lens voltage −25.00 (V); Skimmer voltage −15.00 (V); Inject Flatopole Offset −8.00 (V); and Bent Flatapole DC −6.00 (V). For negative mode ionization: source voltage −3.8 kV.

The calibration mass range was extended to cover small metabolites by inclusion of low-mass calibrants with the standard Thermo calmix masses (below *m*/*z* 138), butylamine (C4H11N1) for positive ion electrospray ionization mode (*m*/*z* 74.096426), and COF3 for negative ion electospray ionization mode (*m*/*z* 84.9906726). To enhance calibration stability, lock-mass correction was also applied to each analytical run. Positive Mode Lock masses, number = 3: Lock Mass #1 (*m*/*z*) 83.0604; Lock Mass #2 (*m*/*z*) 149.0233; and Lock Mass #3 (*m*/*z*) 445.1200. Negative Mode Lock masses, number = 1: Lock Mass #1 (*m*/*z*) 89.0244. For data processing, instrument raw files were converted to positive and negative ionization mode mzXML files. These files were then analyzed using the XCMS/MZMatch/IDEOM pipeline to produce the IDEOM file^58–60^.

### Growth at 28 °C

To establish growth kinetics at 28 °C, overnight cultures were adjusted to an OD_600nm_ of 1 and serially diluted in TSB. A volume of 5 μl of diluted cultures were spotted onto TSA plates supplemented with the appropriate antibiotics if necessary, and plates were incubated at 28 °C for 24 h. Representative pictures were taken.

### Triton resistance test

Triton gradient agar plates (0–0.5%) were used to compare the resistance phenotype against Triton X-100 (USB)^61^. OmniTray single-well plates (NUNC) were filled with TSA 0.5% (v/v) Triton X-100, and a slope was created when the media was still in liquid state. After media solidification, plates were totally filled with TSA, generating the triton gradient. Overnight cultures of the strains were diluted in TSB to an OD_600nm_ = 0.4 and cell suspensions were swabbed across the agar plates containing triton concentration gradients. Plates were incubated at 37 °C for 24 h. Triton sensitivity was observed as a growth deficiency on the concentrated area of the plate.

### Antibiotic resistance

Antimicrobial susceptibility tests were performed at the University Clinic of Navarra. Antibiotics routinely used in clinic for the treatment of *S. aureus* infections were tested using VITEK 2 system (bioMérieux) according to the manufacturer’s instructions.

### Mouse infection models

Strains were cultured overnight in TSA plates at 37 °C and a single colony was resuspended into 5 ml of PBS to an OD_600nm_ = 0.2 (1 × 10^8^ colony-forming units (CFU) per ml). For the kidney colonization assays, 5-week-old CD1 female mice (Charles-River) were inoculated by eye vein injection with 100 µl (1 × 10^7^ cells) of these diluted suspensions of *S. aureus*. Groups of seven mice were used for each strain tested. After 1 week, mice were euthanized and kidneys were removed. Pictures of the kidneys were taken to show abscess formation and then the organs were homogenized in PBS (9 ml g^−1^), serial dilutions were performed and spread on TSA plates in triplicate for determination of the number of CFU per organ gram. For survival assays, 5-week-old CD1 female mice were inoculated by eye vein injection with 100 µl (1 × 10^7^ cells) of diluted suspensions of *S. aureus*. Groups of seven mice were used for each strain tested. Mice death was monitored every day for 7 weeks.

Animal studies were performed in accordance with the European Community guiding in the care and use of animals (Directive 2010/63/EU). Protocols were approved by the ethics committee of the Public University of Navarra (“Comité de Ética, Experimentación Animal y Bioseguridad” of the Universidad Pública de Navarra). Work was carried out in the animal facility of the Instituto de Agrobiotecnología, Universidad Pública de Navarra. Animals were housed under controlled environmental conditions with food and water ad libitum. Mice were euthanized by CO_2_ inhalation followed by cervical dislocation and all efforts were made to minimize suffering.

In these animal studies randomization methods were not used, and authors were not blinded to the group allocation during the experiment and when assessing the outcome.

### Protein A expression analysis

To analyze protein A expression in CDM and RPMI media, overnight cultures were adjusted to an OD_600nm_ of 0.1, diluted 1/40 in CDM or RPMI media, respectively, and incubated at 37 °C without shaking for 24 h in sterile 24-well polystyrene microtiter plates (Sarstedt). A volume of 12 ml of these bacterial cultures were centrifuged and pellets were washed with PBS and resuspended in 120 µl PBS. Then, 1 µl of lysostaphin 10 mg ml^−1^ (Sigma) and 1 µl of TURBO DNase 2 units per ml (Ambion) were added. After 5 h of incubation at 37 °C, cell lysates were centrifuged and supernatants were collected. Protein concentration was determined with the Bio-Rad protein assay (Bio-Rad). Samples were adjusted to 3–5 µg of total protein and one volume of Laemmli buffer was added. Protein extracts were denatured by boiling at 100 °C for 5 min. Proteins were separated on 12% SDS-polyacrylamide gels and stained with 0.25% Coomassie brilliant blue R250 (Sigma) or Criterion TGX Stain-Free Precast Gels (Bio-Rad Cat#5678044). For western blotting, proteins were transferred onto Hybond-ECL nitrocellulose membranes (Amersham Biosciences) by semi-dry electroblotting. Membranes were blocked overnight with 5% skimmed milk in PBS with 0.1% Tween 20, and incubated with goat anti-mouse secondary antibodies labeled with horseradish peroxidase (Sigma) diluted 1:2500 for 1 h at room temperature. Protein A was detected with the SuperSignal West Pico Chemiluminescent Substrate (Thermo Fisher Scientific). To analyze protein A expression in TSB medium, overnight cultures were grown in TSB medium at 37 °C with shaking at 200 rpm. A volume of 5 ml of bacterial cultures were centrifuged and pellets were washed with PBS and resuspended in 120 µl PBS. Then, cells were lysed and protein extracts were prepared as described above. Protein A expression was detected as described above. Densitometry quantification of the protein A western blotting bands from MW2 and Δ*arlS* strains growing in the presence or absence of colistin was determined using ImageJ program. Ratios of the protein A band intensity from the strains growing in the presence or absence of colistin were calculated dividing the protein A band intensity from the strain growing in the presence of colistin by the protein A band intensity from the strain growing in absence of colistin. The average and SD of three independent assays were recorded.

### RNA extractions

To analyze the *spa* RNA levels in CDM medium, overnight cultures were adjusted to an OD_600nm_ of 0.1, diluted 1/40 in CDM medium, and incubated at 37 °C without shaking for 24 h in sterile 24-well polystyrene microtiter plates (Sarstedt). A volume of 12 ml of these bacterial cultures were centrifuged and pellets were frozen in liquid nitrogen and stored at −80 °C until needed.

To analyze the *spa* RNA levels in TSB medium, overnight cultures were grown in TSB medium at 37 °C with shaking at 200 rpm. A volume of 5 ml of bacterial cultures were centrifuged, the pellets were frozen in liquid nitrogen, and stored at −80 °C until needed. Total RNA from bacterial pellets was extracted using the TRIzol reagent method as described^62^. RNA concentrations were quantified and RNAs were stored at −80 °C until needed.

### Real-time quantitative PCR experiments

The *spa* RNA was quantified by real-time quantitative PCR (RT-qPCR) using AriaMx Real-Time PCR System and GoTaq 1-Step RT-qPCR System kit (Promega Ref: A6020). *spa* RNA was amplified with *spa*-FW and *spa*-RV primers, and *gyrB* RNA was used as endogenous controls using primers *gyr*-FW and *gyr*-RV^63^ (Supplementary Table 5). The specificity of RT-qPCR products was monitorized through the analysis of melting curves and electrophoresis. Only samples with no *gyrB* amplification of the minus reverse transcriptase aliquot were considered in the study. The amount of *spa* RNA was expressed as 2^−ΔΔCt^, where ΔCt represents the difference in threshold cycle between the target and control (gyrase) genes and ΔΔCt represents the difference in ΔCt between the studying strain and the wild-type strain.

### Hemolysis assay

To analyze hemolysins production, overnight cultures grown in TSB medium at 37 °C were diluted in TSB to an OD_600nm_ of 1, and 5 μl of diluted cultures were spotted onto 5% sheep blood Columbia agar plates (bioMérieux) and 5% rabbit blood TSA plates (TSA from Pronadisa supplemented with defibrinated rabbit blood). Plates were incubated at 37 °C for 24 h. Hemolysins production was observed by the appearance of a clear halo.

### Quantification of nitrite production

Bacterial strains were cultured in TSB media with 20 mM potassium nitrate (KNO_3_) under decreased oxygen tension conditions that were created by growing the bacteria in 15 ml Falcon tubes completely filled with medium^64^. Strains were incubated overnight at 37 °C with shaking (200 rpm) and the presence of nitrites in the media was determined colorimetrically by the Griess Reagent System (Promega) according to the manufacturer’s instructions. A purple/magenta color indicates the presence of nitrites in the media. Representative pictures were taken.

### Construction of plasmids expressing HK and/or RR genes

To construct the plasmids expressing the RRs, we amplified them by PCR using the corresponding forward (Fw) and reverse (Rv) primers (Supplementary Table 5) and the MW2 chromosomal DNA as template. Forward primers carried a *Sal*I-*Xho*I tail and reverse primers carried a *Bam*HI-*Xma*I-*Kpn*I tail. PCR products were amplified with Phusion^®^ High-Fidelity DNA Polymerase (Thermo Fisher Scientific), purified, and cloned in pCR^®^-Blunt II TOPO vector (Invitrogen). Fragments were then ligated using *Sal*I and *Kpn*I enzymes in plasmid pCN51 (P_cad_ promoter)^65^ and electroporated into *E. coli* XL1-Blue (Stratagene) to generate the pCN51::RRn plasmid collection (Supplementary Table 4).

Similarly, HKs were amplified by PCR with the corresponding Fw and Rv primers (Supplementary Table 5) and the MW2 chromosomal DNA as template. Forward primers carried a *Bam*HI tail and reverse primers carried a *Xma*I-*Asc*I tail. PCR products were amplified with Phusion^®^ High-Fidelity DNA Polymerase (Thermo Fisher Scientific), purified, and cloned in pCR^®^-Blunt II TOPO vector (Invitrogen).

For the construction of plasmids expressing a combination of HKs and RRs, TOPO HKn plasmids were digested with *Bam*HI and *Xma*I enzymes, and the *Bam*HI/*Xma*I module, containing the HK, was transferred to the corresponding plasmid pCN51::RRn and electroporated into *E. coli* XL1-Blue to generate the pCN51::RRn-HKn plasmid collection (Supplementary Table 4).

The plasmid expressing ArlR with D52A amino-acid substitution was constructed by amplifying separately two DNA fragments by PCR using ArlR-Fw/ArlR(D52A)-Rv and ArlR(D52A)-Fw/ArlR-Rv primer pairs. These two PCR products were used as templates in an overlapping PCR, using ArlR-Fw/ArlR-Rv primers to amplify the mutant allele. The arlR(D52A) allele was cloned in pCN51 plasmid as we have described above for the construction of plasmids expressing RRs.

The plasmid expressing ArlS with H242A amino-acid substitution was constructed by amplifying separately two DNA fragments by PCR using ArlS-Fw/ArlS(H242A)-Rv and ArlS(H242A)-Fw/ArlS-Rv primer pairs. These two PCR products were used as templates in an overlapping PCR, using ArlS-Fw/ArlS-Rv primers to amplify the mutant allele. The ArlS(H242A) allele was cloned in pCN51::*arlR* plasmid as we have described above for the construction of plasmids expressing HKs and RRs.

Plasmids were purified from *E. coli* XL1-Blue and transformed into *S. aureus* strains by electroporation.

The pCN51 inducible plasmid shows a basal expression in the absence of cadmium. All the experiments performed in this study that involve the pCN51 plasmid were carried out without cadmium supplementation.

### Desiccation experiment

The desiccation experiment was adapted from an already described protocol^66^. Briefly, 1 ml from overnight cultures grown in TSB medium at 37 °C was harvested by centrifugation, washed with PBS three times, and finally resuspended in 1 ml of PBS. The number of viable cells in this initial bacterial suspension was tested immediately (initial numbers), performing serial dilutions and plating dilutions in triplicate, and 100 µl of this initial bacterial suspension were air-dried in 24-well tissue culture plates. Plates were stored at room temperature for 7 days. Dried bacteria were then rehydrated with 500 µl TSB and the number of viable cells remaining in each sample was determined by performing serial dilutions of each sample and plating the dilutions in triplicate. The average and SD of three independent assays were recorded.

### Statistical analysis

All statistical analyses were performed in GraphPad Prism 5.01. Data corresponding to the kidney colonization assay were compared using a two-tailed nonparametric Mann–Whitney test. Data corresponding to the mice survival assay were compared using a log-rank (Mantel–Cox) test. Ratios of protein A quantification by western blot and data from qRT-PCR analysis were statistically analyzed using a two-tailed one-sample *t*-test inferred from a hypothetical value of 1. Finally, data corresponding to desiccation resistance were compared using a two-tailed nonparametric Mann–Whitney test.

### Data availability

Genomic sequence data for strains MW2, MW2 ΔXV, RN1, and RN1 ΔXV have been deposited in the National Center for Biotechnology Information Sequence Read Archive under accession code SRP127351. Differences in the metabolome of *S. aureus* isolate MW2 and ΔXV mutant derivative have been deposited at figshare (10.6084/m9.figshare.5733495.v1). Other relevant data are available in this article and its Supplementary Information files, or from the corresponding author upon request.

## Electronic supplementary material

Supplementary Information

Description of Additional Supplementary Files

Supplementary Data 1
