# Supplementary material for: Sensory deprivation in Staphylococcus aureus
Source: Nat Commun. 2018 Feb 6;9:523. doi: 10.1038/s41467-018-02949-y (PMC5802764; doi:10.1038/s41467-018-02949-y)
Supplement: Supplementary file 1 — Supplementary Information [file 41467_2018_2949_MOESM1_ESM.pdf]

SUPPLEMENTARY FIGURES

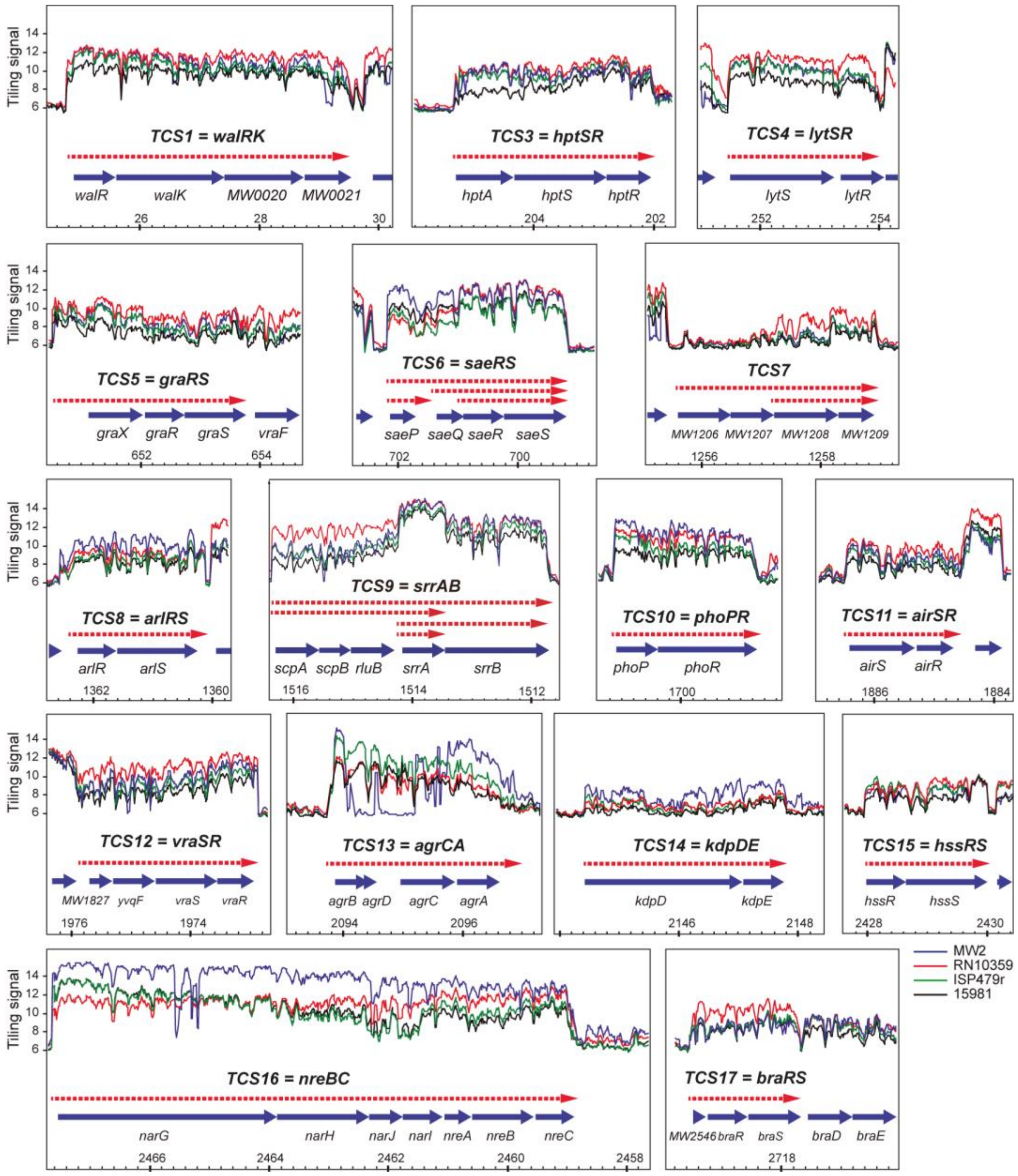

Supplementary Figure 1. **Transcriptomic analysis of the *S. aureus* two-component systems**. Transcriptomic maps showing expression of the TCS family in four genetically unrelated *S. aureus* strains (MW2, RN10359, ISP479r and 15981). Total RNA was purified from bacteria grown in TSB at 37ºC under shaking conditions (250 rpm) until cultures reached an OD_{600} of 0.8. Drawings are images from IGB software showing the regions of the *S. aureus* genome that encode the TCSs operons. Genomic coordinates denote the position in kilobases of the *S. aureus* NCTC 8325 genome. Annotated open reading frames (ORFs) are shown as blue arrows. Numbers or names on the ORF indicate the gene identification. Transcripts are represented as dashed red arrows. The scale indicates $\log_2$ of the tiling signal.

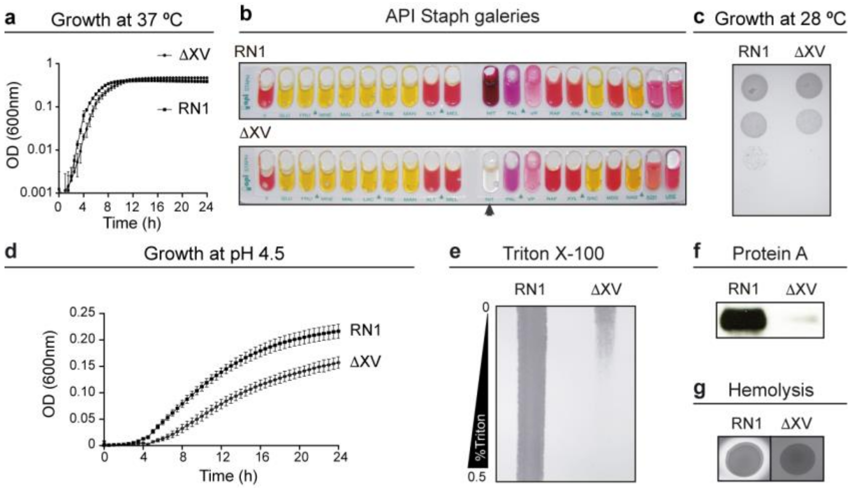

Supplementary Figure 2. **Phenotypic analysis of *S. aureus* RN1 ΔXV strain. a**, Growth curves in TSB medium at 37 ºC. Average and SD of three independent assays are represented. RN1 doubling time: 35 min; ΔXV doubling time: 43 min. **b**, Standard metabolic pathways analyzed using commercial API Staph galleries. Only the capacity to reduce nitrate to nitrite was affected when WT and ΔXV strains were compared (arrowhead). **c**, Bacterial growth on TSA medium at 28 ºC. Serial dilutions were spotted on agar plates. **d**, Growth curves in TSB medium at pH 4.5. Average and SD of three independent assays are represented. **e**, Growth capacity on Triton X-100 concentration gradient agar plates. **f**, Protein A expression in CDM detected by western blotting (full blot is shown in Supplementary Fig. 11). **g**, Hemolysins production on sheep blood agar plates.

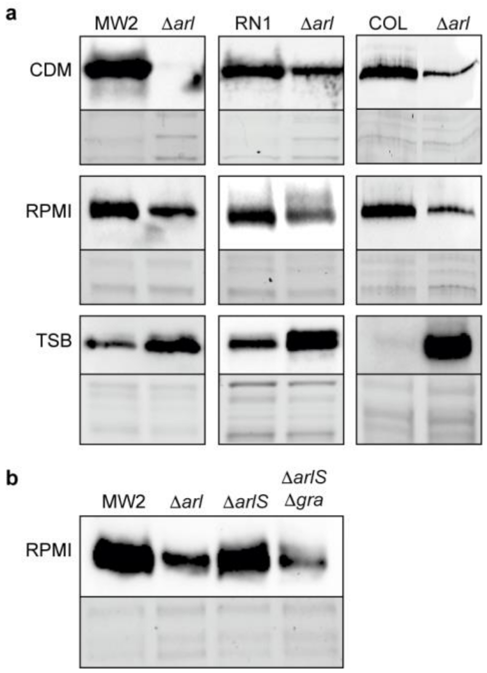

Supplementary Figure 3. Expression of protein A in different *S. aureus* strains grown in different media. a, Protein A expression in *S. aureus* MW2, RN1 and COL strains and their corresponding Δ*arl* mutants grown in CDM, RPMI and TSB media was detected by western blotting. Stain-free gels are shown as a loading control in all sections. **b**, *In vivo* cross-talk analysis between GraS and ArlR in RPMI medium. Protein A expression was detected by western blotting on the following strains: *S. aureus* MW2 and its corresponding Δ*arl*, Δ*arlS* and Δ*arlS* Δ*gra* strains. Stain-free precast gels are shown as a loading control in all sections. Full blots and gels are shown in Supplementary Fig. 12.

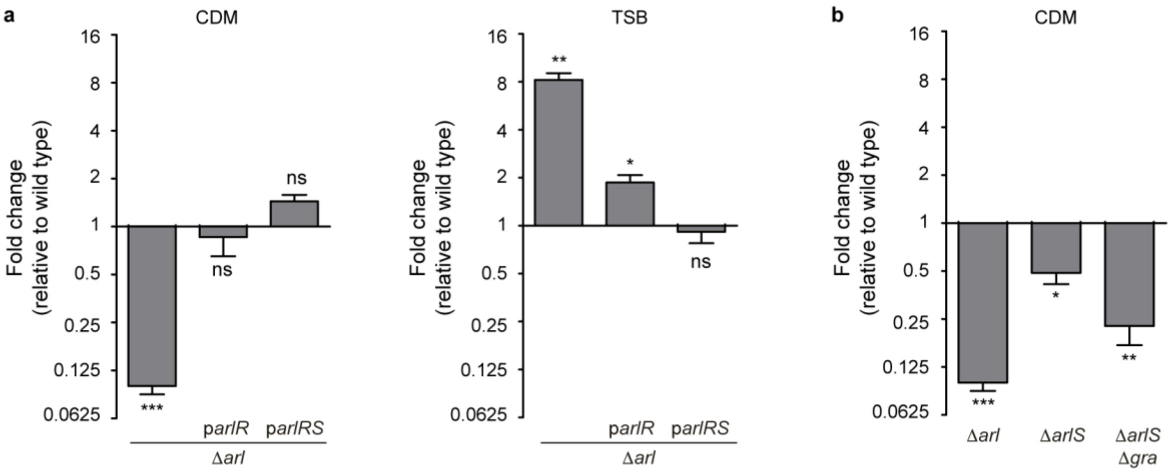

Supplementary Figure 4. Quantitative RT-PCR analyses of *spa* transcript levels. a, *spa* RNA levels in the MW2 wild type strain, the Δ*arl* mutant and the Δ*arl* mutant complemented with *arlR* and *arlRS* grown in CDM or TSB media. **b**, *spa* RNA levels in the MW2 wild type strain and Δ*arl*, Δ*arlS* and Δ*arlS* Δ*gra* mutant strains grown in CDM media. The data are compiled from n=3 independent determinations of *gyrB* RNA normalized cycle thresholds and are presented as $2^{-\Delta\Delta C_T}$, where $\Delta\Delta C_T$ represents the difference in ΔC_T between the studying strain and the MW2 wild type strain. Error bars reflect ± SEM for the normalized C_T values. Note the $\log_2$ scale in the y axis. P-values were determined by a Student *t* test. ns = no significant difference; *p < 0.05; **p < 0.01; ***p<0.001.

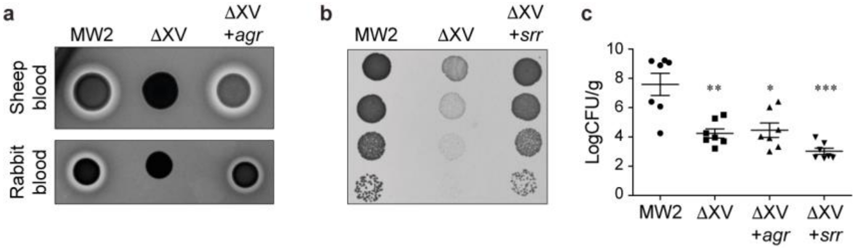

Supplementary Figure 5. Virulence associated phenotypes of ∆XV derivatives containing a restored chromosomal copy of selected TCSs. a, Phenotype rescue in the ∆XV derivative strain containing a restored chromosomal copy of *agr*. Chromosomal restoration of *agr* in ΔXV rescues the capacity to produce hemolysis on sheep and rabbit blood agar plates. **b**, Phenotype rescue in the ∆XV derivative strain containing a restored chromosomal copy of *srr*. Chromosomal restoration of *srr* in ΔXV rescues the capacity to grow on TSA medium at 28 ºC. Serial dilutions were spotted on TSA plates. **c**, Kidney colonization of ∆XV derivative strains containing a chromosomal copy of *agr* or *srr*. Groups of seven CD1 mice were inoculated by eye vein injection with *S. aureus* MW2, ΔXV, ΔXV+*agr* and ΔXV+*srr* strains. After one week, mice were euthanized and kidneys were removed, homogenized and the number of colony-forming units per organ gram was determined. Data were compared using the Mann-Whitney test (n=7, *$p < 0.05$; ** $p< 0.01$; *** $p< 0.001$).

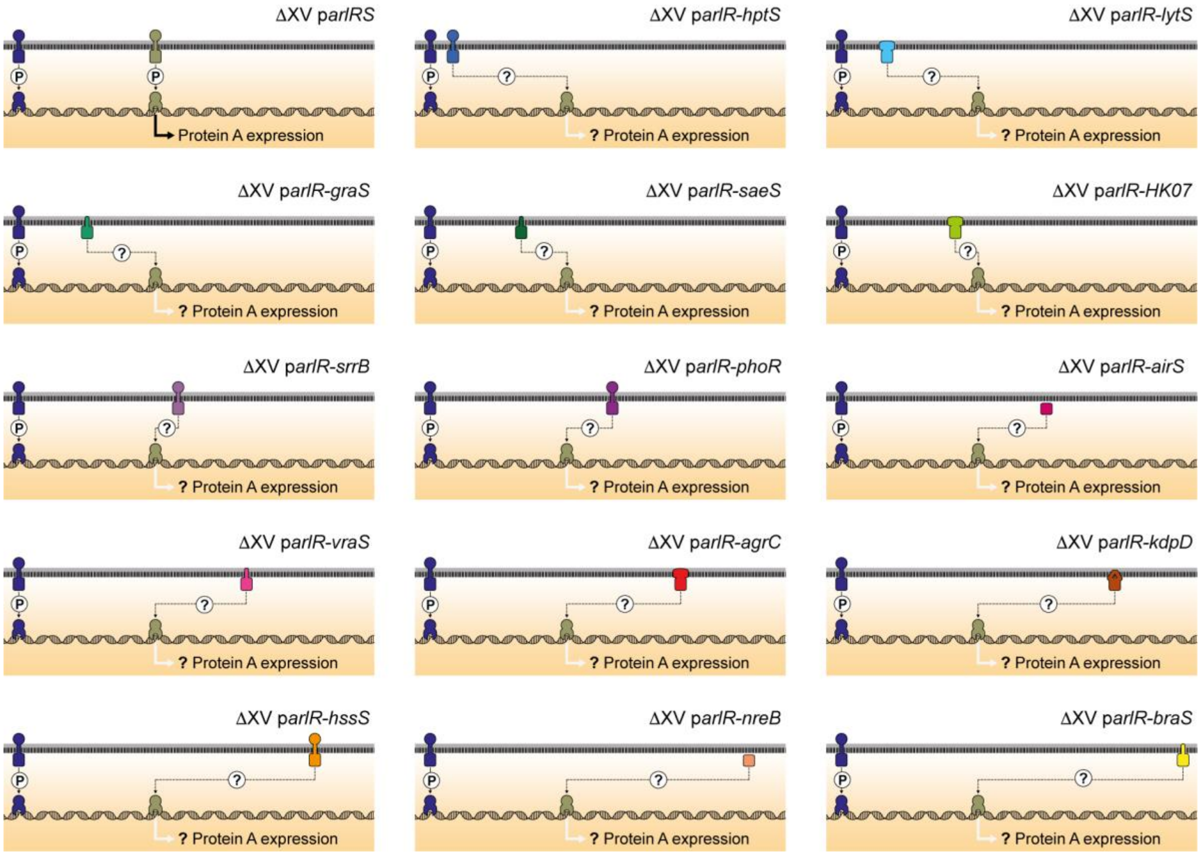

Supplementary Figure 6. **The schematics show the approach used to systematically analyze *in vivo* cross-talk**. A set of fifteen plasmids each containing the *arlR* gene (RR) in combination with each histidine kinase (HK) was used to complement *S. aureus* MW2 ΔXV strain. Protein A expression was the phenotype used to analyze *in vivo* cross-talk.

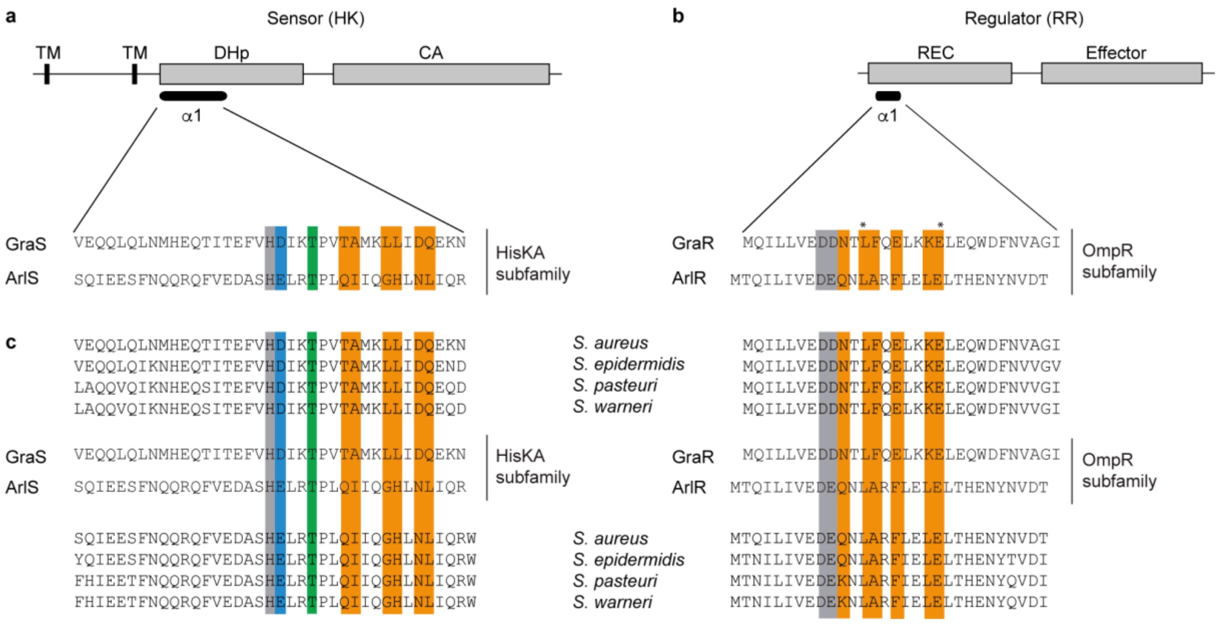

Supplementary Figure 7. Comparative analysis of the amino acids that confer specificity to activate a particular partner in Gra and Arl TCSs. **a**, Specificity residues in the DHp domain of the GraS and ArlS histidine kinases. Based on the DHp domain both HKs are members of the HisKA subfamily. Conserved histidine is coloured grey. Residues required for kinase and phosphatase activities are colored blue and green, respectively. Coevolving specificity residues are colored orange. **b**, Specificity residues in the REC domain of the GraR and ArlR response regulators. Based on the Effector domain both RRs are members of the OmpR/PhoB winged-helix subfamily. Conserved residues are colored grey. Coevolving specificity residues are colored orange and those conserved in both proteins are indicated with an asterisk. **c**, Schematic representation of the specificity residues in the DHp domain of the GraS and ArlS histidine kinases and REC domain of the GraR and ArlR response regulators in different staphylococcal species.

i

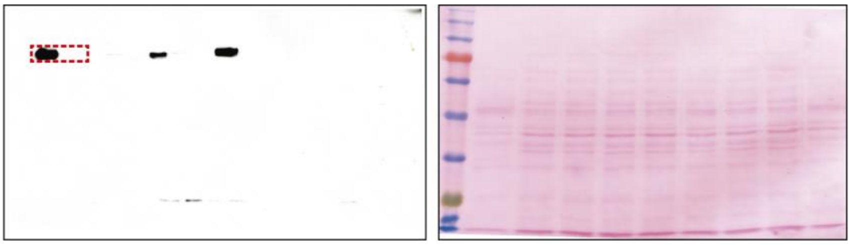

Supplementary Figure 8. Uncropped figure 2i. Ponceau stained membrane is shown as a loading control.

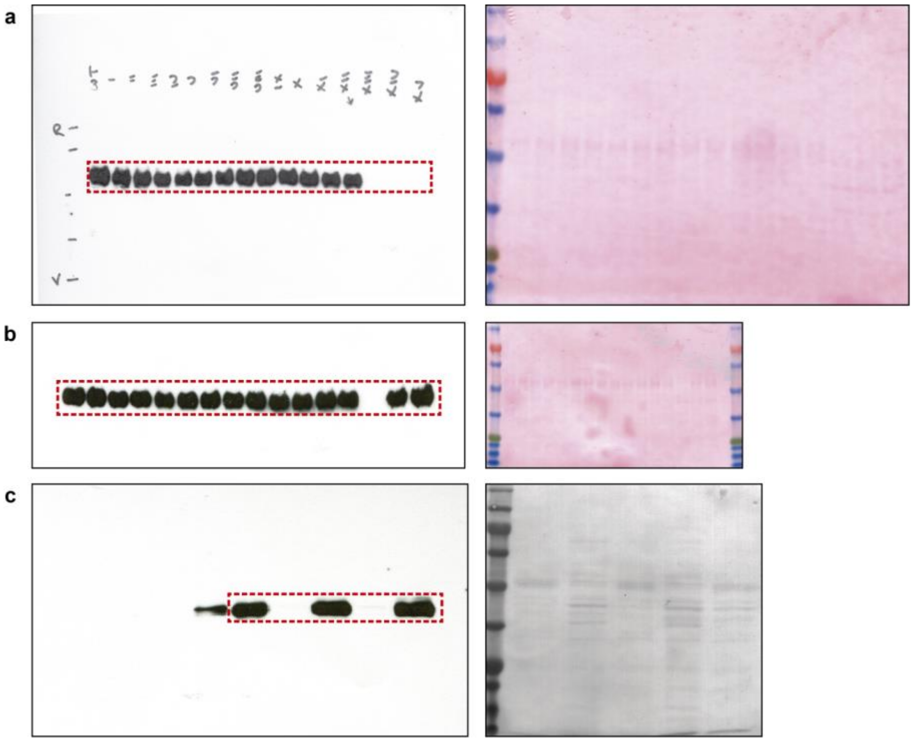

Supplementary Figure 9. Uncropped figure 3: a)iii, b)iii and c)iii sections. Ponceau stained membranes and coomassie stained gel are shown as a loading control.

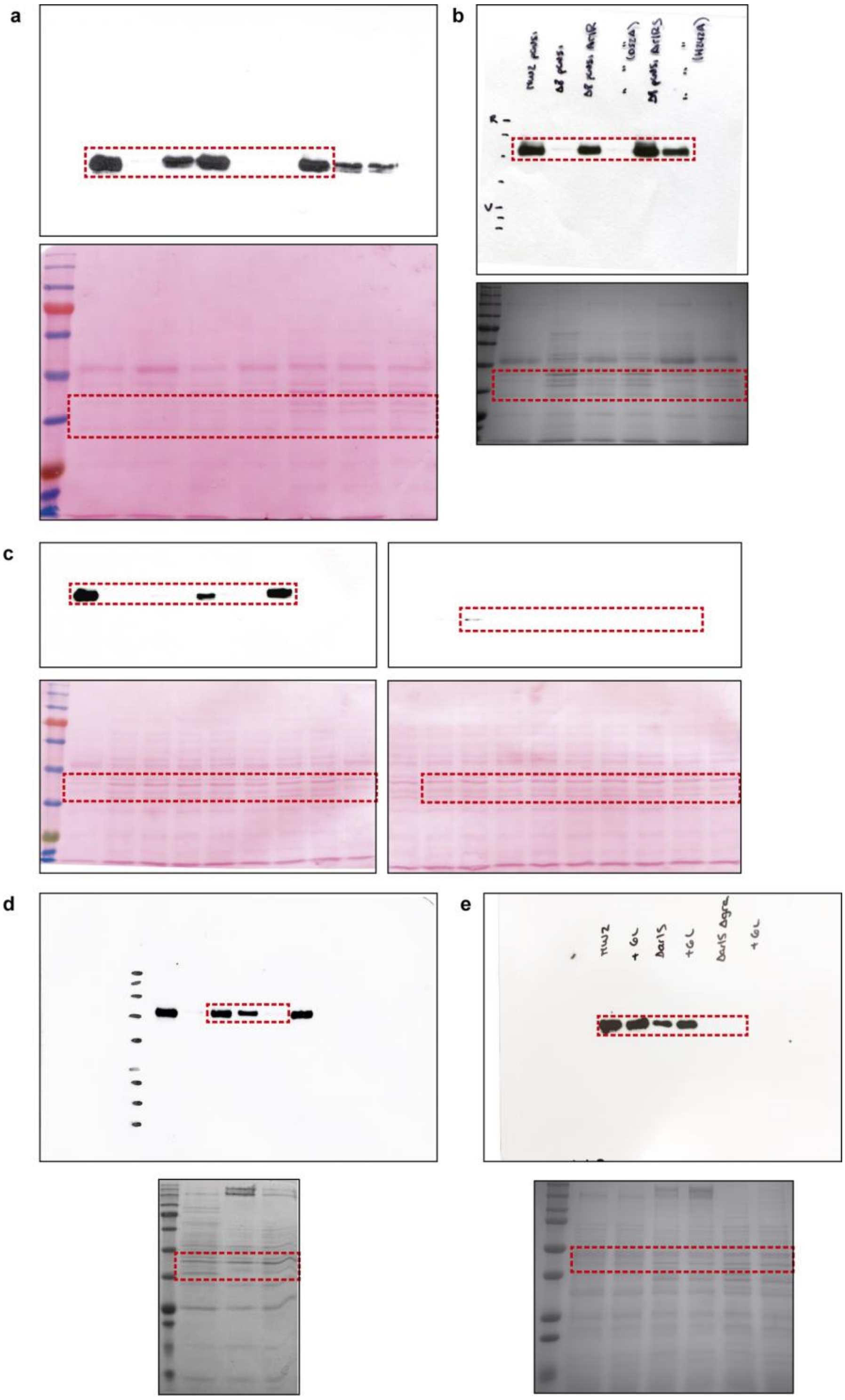

Supplementary Figure 10. Uncropped figure 6: a, b, c, d and e sections.

f

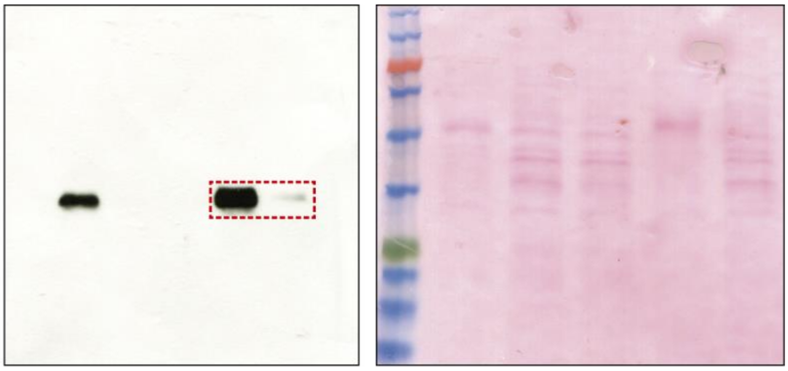

Supplementary Figure 11. Uncropped Supplementary figure 2f. Ponceau stained membrane is shown as a loading control.

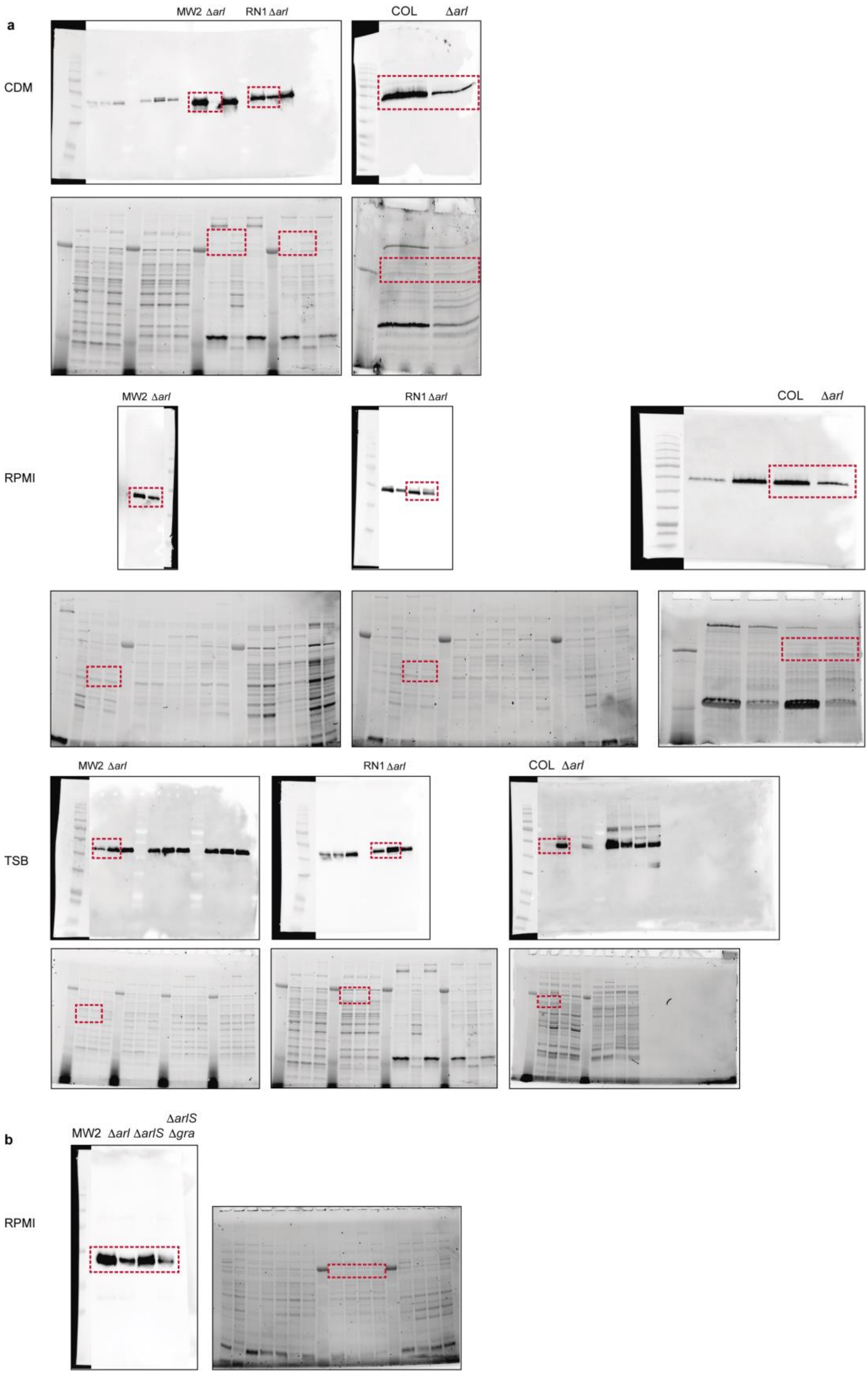

Supplementary Figure 12. Uncropped Supplementary figure 3.

SUPPLEMENTARY TABLES

Supplementary Table 1. Spontaneous mutations generated during the construction of *S. aureus* MW2 ΔXV and *S. aureus* RN1 ΔXV strains.

		***S. aureus* MW2**		
Position	Mutation	Annotation	Gene	Description
239,826	G→A	A220T (**G**CA→**A**CA)	*pflA* →	Formate acetyltransferase activating enzyme
379,604	G→T	G249V (G**G**A→G**T**A)	*MW0330* →	Hypothetical protein
746,147	G→T	E123* (**G**AA→**T**AA)	*nagA* →	N-acetylglucosamine-6-phosphate deacetylase
1,021,368	G→T	D263Y (**G**AT→**T**AT)	*menB* →	Naphthoate synthase
1,105,909	Ω43545 bp	Phage insertion	*rpmF* → / ← *isdB*	50S Ribosomal protein L3 / hypothetical protein
1,267,878	G→T	R475L (C**G**T→C**T**T)	*pnpA* →	Polynucleotide phosphorylase / polyadenylase
1,283,489	G→C	G502A (G**G**T→G**C**T)	*MW1169* →	Phosphodiesterase
1,691,188	G→T	L10I (**C**TA→**A**TA)	*MW1565* ←	Hypothetical protein
2,639,004	GT→TG	Y441S (T**AC**→T**CA**)	*rocA* ←	1-Pyrroline-5-carboxylate dehydrogenase
2,639,012	T→A	T439S (**A**CA→**T**CA)	*rocA* ←	1-Pyrroline-5-carboxylate dehydrogenase
		***S. aureus* RN1**		
Position	Mutation	Annotation	Gene	Description
781,629	C→T	Y258Y (TA**C**→TA**T**)	*eno* →	Phosphopyruvate hydratase
787,913	C→T	intergenic (+907/+415)	*smpB* → / ← *SAOUHSC_00806*	SsrA‑binding protein / hypothetical protein
1,101,720	A→G	intergenic (+149/-237)	*SAOUHSC_01150* → / → *SAOUHSC_01152*	Cell division protein FtsZ / hypothetical protein
1,560,503	C→T	A190T (**G**CT→**A**CT)	*SAOUHSC_01646* ←	Glucokinase
1,703,050	T→C	intergenic (-589/-23)	*SAOUHSC_01802* ← / → *SAOUHSC_01803*	Hypothetical protein / hypothetical protein

Supplementary Table 2. Antimicrobial susceptibility testing of wild type and ΔXV strains to antibiotics routinely used for the treatment of *S. aureus* infections.

Antibiotics	MW2	MW2 ΔXV	RN1	RN1 ΔXV
Bencylpenicillin	R	R	R	S
Ampicillin	R	R	R	S
Cloxacillin	R	R	S	S
Oxacillin	R	R	S	S
Cefalotin	R	R	S	S
Cefuroxime	R	R	S	S
Gentamicin	S	S	S	S
Tobramycin	S	S	S	S
Ciprofloxacin	S	S	S	S
Levofloxacin	S	S	S	S
Inducible resistance to Clindamycin	-	-	-	-
Azithromycin	S	S	S	S
Clarithromycin	S	S	S	S
Erithromycin	S	S	S	S
Clindamycin	S	S	S	S
Quinuspristin/Dalfopristin	S	S	S	S
Linezolid	S	S	S	S
Teicoplanin	S	S	S	S
Vancomycin	S	S	S	S
Tigecycline	S	S	S	S
Fosfomycin	S	S	S	S
Nitrofurantoin	S	S	S	S
Fusidic acid	S	S	S	S
Mupirocin	S	S	S	S
Rifampicin	S	S	S	S
Trimethoprim/Sulfamethoxazole	S	S	S	S
Cefoxitin	R	S	S	S
Chloramphenicol	S	S	S	S
MRSA	+	+	-	-

R: Resistant; S: Susceptible; MRSA: Methicillin-resistant *Staphylococcus aureus*.
+ or - indicate the presence or absence of the corresponding feature.

Supplementary Table 3. Strains used in this study.

Strains	Relevant characteristics	MIC*	Reference
Staphylococcus aureus			
15981	Clinical isolate	532	[1]
RN10359	RN450 lysogenic for 80α phage	3337	[2]
ISP479r	ISP479c with *rsbU* gene restored	1680	[3]
RN4220	A mutant of 8325-4 *S. aureus* strain that accepts foreign DNA	99	[4]
COL	Wild type strain	6509	[5]
COL Δ*arl*	COL Δ*arlRS*	6510	[5]
MW2	Typical community-acquired strain of MRSA, which was isolated in 1998 in North Dakota, USA.	3566	[6]
Δ*hpt*	MW2 Δ*hptRS*	4032	This study
Δ*lyt*	MW2 Δ*lytSR*	2964	This study
Δ*gra*	MW2 Δ*graRS*	11	This study
Δ*sae*	MW2 Δ*saeRS*	2965	This study
Δ*tcs7*	MW2 Δ*MW1208-MW1209*	4033	This study
Δ*arl*	MW2 Δ*arlRS*	4034	This study
Δ*srr*	MW2 Δ*srrAB*	2966	This study
Δ*pho*	MW2 Δ*phoPR*	4035	This study
Δ*air*	MW2 Δ*airSR*	3670	This study
Δ*vra*	MW2 Δ*vraSR*	4036	This study
Δ*agr*	MW2 Δ*agrBDCA*	4037	This study
Δ*kdp*	MW2 Δ*kdpDE*	4038	This study
Δ*hss*	MW2 Δ*hssRS*	2979	This study
Δ*nre*	MW2 Δ*nreBC*	2967	This study
Δ*bra*	MW2 Δ*braRS*	4039	This study
ΔI	MW2 Δ*airSR*	3670	This study
ΔII	MW2 ΔI Δ*hptRS*	3713	This study
ΔIII	MW2 ΔII Δ*lytSR*	3717	This study
ΔIV	MW2 ΔIII Δ*graRS*	66	This study
ΔV	MW2 ΔIV Δ*saeRS*	371	This study
ΔVI	MW2 ΔV Δ*MW1208-MW1209*	768	This study
ΔVII	MW2 ΔVI Δ*hssRS*	1336	This study
ΔVIII	MW2 ΔVII Δ*nreBC*	1345	This study
ΔIX	MW2 ΔVIII Δ*braRS*	1379	This study
ΔX	MW2 ΔIX Δ*kdpDE*	2955	This study
ΔXI	MW2 ΔX Δ*vraSR*	2956	This study
ΔXII	MW2 ΔXI Δ*phoPR*	2957	This study
ΔXIII	MW2 ΔXII Δ*arlRS*	2958	This study
ΔXIV	MW2 ΔXIII Δ*agrBDCA*	2960	This study
ΔXV	MW2 ΔXIV Δ*srrAB*	2961	This study
RN1	8325 with a restored *rsbU* gene	2968	R. Novick
RN1 ΔI	RN1 Δ*airSR*	3999	This study
RN1 ΔII	RN1 ΔI Δ*lytSR*	4000	This study
RN1 ΔIII	RN1 ΔII Δ*graRS*	4001	This study
RN1 ΔIV	RN1 ΔIII Δ*saeRS*	4002	This study
RN1 ΔV	RN1 ΔIV Δ*SAOUHSC_01313-SAOUHSC_01314*	4003	This study
RN1 ΔVI	RN1 ΔV Δ*hptRS*	4004	This study
RN1 ΔVII	RN1 ΔVI Δ*arlRS*	4005	This study
RN1 ΔVIII	RN1 ΔVII Δ*srrAB*	4006	This study
RN1 ΔIX	RN1 ΔVIII Δ*phoPR*	4007	This study
RN1 ΔX	RN1 ΔIX Δ*hssRS*	4008	This study
RN1 ΔXI	RN1 ΔX Δ*nreBC*	4009	This study
RN1 ΔXII	RN1 ΔXI Δ*braRS*	4010	This study
RN1 ΔXIII	RN1 ΔXII Δ*vraSR*	4011	This study
RN1 ΔXIV	RN1 ΔXIII Δ*kdpDE*	4012	This study
RN1 ΔXV	RN1 ΔXIV Δ*agrBDCA*	2969	This study
ST1000	RN4220 P*spac::walRK* (*walRK* operon under the *spac* promoter control through the integration of pSD3-3 plasmid)	2970	[7]
MW2 P*spac::walRK*	MW2 with the *walRK* operon under the *spac* promoter control through the integration of pSD3-3 plasmid	2973	This study
MW2 ΔXVI*	MW2 ΔXV P*spac::walRK* (*walRK* operon under the *spac* promoter control through the integration of pSD3-3 plasmid)	2975	This study
RN1 P*spac::walRK*	RN1 with the *walRK* operon under the *spac* promoter control through the integration of pSD3-3 plasmid	5608	This study
RN1 ΔXVI*	RN1 ΔXV P*spac::walRK* (*walRK* operon under the *spac* promoter control through the integration of pSD3-3 plasmid)	5609	This study
RN1 Δ*arl*	RN1 Δ*arlRS*	6260	This study
ΔXV p*arlR*	MW2 ΔXV carrying pCN51::*arlR* plasmid	4891	This study
ΔXV p*arlRS*	MW2 ΔXV carrying pCN51::*arlRS* plasmid	4538	This study
Δ*arl* p*arlR*	MW2 Δ*arl* carrying pCN51::*arlR* plasmid	4890	This study

Continued on the following page

Supplementary Table 3. Continued.

Strains	Relevant characteristics	MIC*	Reference
Δ*arl* p*arlRS*	MW2 Δ*arl* carrying pCN51::*arlRS* plasmid	4539	This study
ΔXV p*vraR*	MW2 ΔXV carrying pCN51::*vraR* plasmid	4708	This study
ΔXV p*vraRS*	MW2 ΔXV carrying pCN51::*vraRS* plasmid	4676	This study
Δ*vra* p*vraR*	MW2 Δ*vra* carrying pCN51::*vraR* plasmid	4889	This study
Δ*vra* p*vraRS*	MW2 Δ*vra* carrying pCN51::*vraRS* plasmid	4675	This study
ΔXV p*srrA*	MW2 ΔXV carrying pCN51::*srrA* plasmid	4678	This study
ΔXV p*srrAB*	MW2 ΔXV carrying pCN51::*srrAB* plasmid	4680	This study
Δ*srr* p*srrA*	MW2 Δ*srr* carrying pCN51::*srrA* plasmid	4677	This study
Δ*srr* p*srrAB*	MW2 Δ*srr* carrying pCN51::*srrAB* plasmid	4679	This study
ΔXV p*nreC*	MW2 ΔXV carrying pCN51::*nreC* plasmid	3888	This study
ΔXV p*nreCB*	MW2 ΔXV carrying pCN51::*nreCB* plasmid	4536	This study
Δ*nre* p*nreC*	MW2 Δnre carrying pCN51::*nreC* plasmid	3889	This study
Δ*nre* p*nreCB*	MW2 Δnre carrying pCN51::*nreCB* plasmid	4535	This study
ΔXV p*graR*	MW2 ΔXV carrying pCN51::*graR* plasmid	5718	This study
ΔXV p*graRS*	MW2 ΔXV carrying pCN51::*graRS* plasmid	5270	This study
Δ*gra* p*graR*	MW2 Δ*gra* carrying pCN51::*graR* plasmid	5716	This study
Δ*gra* p*graRS*	MW2 Δ*gra* carrying pCN51::*graRS* plasmid	5717	This study
Δ*arl* pCN51	MW2 Δ*arl* carrying pCN51 empty plasmid	4704	This study
Δ*vra* pCN51	MW2 Δ*vra* carrying pCN51 empty plasmid	4684	This study
Δ*srr* pCN51	MW2 Δ*srr* carrying pCN51 empty plasmid	4683	This study
Δ*nre* pCN51	MW2 Δ*nre* carrying pCN51 empty plasmid	4705	This study
Δ*gra* pCN51	MW2 Δ*gra* carrying pCN51 empty plasmid	5715	This study
MW2 pCN51	MW2 carrying pCN51 empty plasmid	4681	This study
ΔXV pCN51	MW2 ΔXV carrying pCN51empty plasmid	4682	This study
ΔXV p*arlR-hptS*	MW2 ΔXV carrying pCN51::*arlR-hptS* plasmid	5041	This study
ΔXV p*arlR-lytS*	MW2 ΔXV carrying pCN51::*arlR-lytS* plasmid	5042	This study
ΔXV p*arlR-graS*	MW2 ΔXV carrying pCN51::*arlR-graS* plasmid	5043	This study
ΔXV p*arlR-saeS*	MW2 ΔXV carrying pCN51::*arlR-saeS* plasmid	5044	This study
ΔXV p*arlR-HK07*	MW2 ΔXV carrying pCN51::*arlR-HK07* plasmid	5045	This study
ΔXV p*arlR-srrB*	MW2 ΔXV carrying pCN51::*arlR-srrA* plasmid	5046	This study
ΔXV p*arlR-phoR*	MW2 ΔXV carrying pCN51::*arlR-phoR* plasmid	5047	This study
ΔXV p*arlR-airS*	MW2 ΔXV carrying pCN51::*arlR-airS* plasmid	5048	This study
ΔXV p*arlR-vraS*	MW2 ΔXV carrying pCN51::*arlR-vraS* plasmid	5049	This study
ΔXV p*arlR-agrC*	MW2 ΔXV carrying pCN51::*arlR-agrC* plasmid	5050	This study
ΔXV p*arlR-kdpD*	MW2 ΔXV carrying pCN51::*arlR-kdpD* plasmid	5051	This study
ΔXV p*arlR-hssS*	MW2 ΔXV carrying pCN51::*arlR-hssS* plasmid	5052	This study
ΔXV p*arlR-nreB*	MW2 ΔXV carrying pCN51::*arlR-nreB* plasmid	5053	This study
ΔXV p*arlR-braS*	MW2 ΔXV carrying pCN51::*arlR-braS* plasmid	5054	This study
Δ*arl* p*arlR*(D52A)	MW2 Δ*arl* carrying pCN51::*arlR*(D52A) plasmid	5001	This study
Δ*arl* p*arlRS*(H242A)	MW2 Δ*arl* carrying pCN51::*arlRS*(H242A) plasmid	4924	This study
Δ*arlS*	MW2 Δ*arlS*	5438	This study
Δ*arlS* Δ*gra*	MW2 Δ*arlS* Δ*graRS*	5537	This study
ΔXV+*agr*	MW2 ΔXV with restored *agrBDCA* genes in the chromosome	6601	This study
ΔXV+*srr*	MW2 ΔXV with restored *srrAB* genes in the chromosome	6602	This study

*Microbial Pathogenesis Laboratory strain collection number

Supplementary Table 4. Plasmids used in this study.

Plasmids	Relevant characteristics	Reference
pMAD	*E. coli - S. aureus* shuttle vector with a thermosensitive origin of replication for Gram-positive bacteria. The vector contains the *bgaB* gene encoding a ß- galactosidase under the control of a constitutive promoter as reporter of plasmid presence. Ap^R, Em^R.	[8]
pMAD::TCS3AD	pMAD plasmid containing the allele for deletion of the *hptRS* genes	This study
pMAD::TCS4AD	pMAD plasmid containing the allele for deletion of the *lytSR* genes	This study
pMAD::TCS5AD	pMAD plasmid containing the allele for deletion of the *graRS* genes	This study
pMAD::TCS6AD	pMAD plasmid containing the allele for deletion of the *saeRS* genes	[3]
pMAD::TCS7AD	pMAD plasmid containing the allele for deletion of the *MW1208-MW1209* genes	This study
pMAD::TCS8AD	pMAD plasmid containing the allele for deletion of the *arlRS* genes	[3]
pMAD::TCS9AD	pMAD plasmid containing the allele for deletion of the *srrAB* genes	This study
pMAD::TCS10AD	pMAD plasmid containing the allele for deletion of the *phoPR* genes	[3]
pMAD::TCS11AD	pMAD plasmid containing the allele for deletion of the *airSR* genes	This study
pMAD::TCS12AD	pMAD plasmid containing the allele for deletion of the *vraSR* genes	[3]
pMAD::TCS13AD	pMAD plasmid containing the allele for deletion of the *agrBDCA* genes	[1]
pMAD::TCS14AD	pMAD plasmid containing the allele for deletion of the *kdpDE* genes	This study
pMAD::TCS14AD(RN1)	pMAD plasmid containing the allele for deletion of the *kdpDE* genes from RN1	This study
pMAD::TCS15AD	pMAD plasmid containing the allele for deletion of the *hssRS* genes	[3]
pMAD::TCS16AD	pMAD plasmid containing the allele for deletion of the *nreBC* genes	This study
pMAD::TCS17AD	pMAD plasmid containing the allele for deletion of the *braRS* genes	This study
pMAD::TCS9	pMAD plasmid containing the allele to restore *srrAB* genes	This study
pMAD::TCS13	pMAD plasmid containing the allele to restore *agrBDCA* genes	This study
pSD3-3	pDH88 plasmid containing a 432-bp fragment, carrying the ribosome-binding site and 5´ portion of *walR*, and used to construct strains with *walRK* operon under the *spac* promoter control	[9]
pCN51	*E. coli - S. aureus* shuttle vector to express genes under the control of the P_{cad} cadmium-inducible promoter. Low copy number (20 to 25 copies/cell). Em^R .	[10]
pCN51::*arlRS*	pCN51 plasmid expressing *arlRS* genes	This study
pCN51::*arlR*	pCN51 plasmid expressing *arlR* gene	This study
pCN51::*srrAB*	pCN51 plasmid expressing *srrAB* genes	This study
pCN51::*srrA*	pCN51 plasmid expressing *srrA* gene	This study
pCN51::*vraRS*	pCN51 plasmid expressing *vraRS* genes	This study
pCN51::*vraR*	pCN51 plasmid expressing *vraR* gene	This study
pCN51::*nreCB*	pCN51 plasmid expressing *nreCB* genes	This study
pCN51::*nreC*	pCN51 plasmid expressing *nreC* gene	This study
pCN51::*graRS*	pCN51 plasmid expressing *graRS* genes	This study
pCN51::*graR*	pCN51 plasmid expressing *graR* gene	This study
pCN51::*arlR-hptS*	pCN51 plasmid expressing *arlR* (RR) and *hptS* (HK) genes	This study
pCN51::*arlR-lytS*	pCN51 plasmid expressing *arlR* (RR) and *lytS* (HK) genes	This study
pCN51::*arlR-graS*	pCN51 plasmid expressing *arlR* (RR) and *graS* (HK) genes	This study
pCN51::*arlR-saeS*	pCN51 plasmid expressing *arlR* (RR) and *saeS* (HK) genes	This study
pCN51::*arlR-HK07*	pCN51 plasmid expressing *arlR* (RR) and *HK07* (HK) genes	This study
pCN51::*arlR-srrB*	pCN51 plasmid expressing *arlR* (RR) and *srrB* (HK) genes	This study
pCN51::*arlR-phoR*	pCN51 plasmid expressing *arlR* (RR) and *phoR* (HK) genes	This study
pCN51::*arlR-airS*	pCN51 plasmid expressing *arlR* (RR) and *airS* (HK) genes	This study
pCN51::*arlR-vraS*	pCN51 plasmid expressing *arlR* (RR) and *vraS* (HK) genes	This study
pCN51::*arlR-agrC*	pCN51 plasmid expressing *arlR* (RR) and *agrC* (HK) genes	This study
pCN51::*arlR-kdpD*	pCN51 plasmid expressing *arlR* (RR) and *kdpD* (HK) genes	This study
pCN51::*arlR-hssS*	pCN51 plasmid expressing *arlR* (RR) and *hssS* (HK) genes	This study
pCN51::*arlR-nreB*	pCN51 plasmid expressing *arlR* (RR) and *nreB* (HK) genes	This study
pCN51::*arlR-braS*	pCN51 plasmid expressing *arlR* (RR) and *braS* (HK) genes	This study
pCN51::*arlR*(D52A)	pCN51 plasmid expressing an ArlR protein with D52A amino acid substitution	This study
pCN51::*arlRS*(H242A)	pCN51 plasmid expressing ArlR protein and ArlS protein with H242A amino acid substitution	This study

Supplementary Table 5. Oligonucleotides used in this study.

Oligonucleotide	Sequence
**Deletion of two-component systems**	
hpt-A (BamHI)	*GGATCC*GTTATCAGAAACAATTGATCA
hpt-B (NcoI)	*CCATGG*TGAATCATCTCCAAAAATTTAT
hpt-C (NcoI)	*CCATGG*ATTAGCACATAACTAATGATTA
hpt-D (XhoI)	*CTCGAG*TAAAGGCGATGCAGTTAATA
lyt-A (BamHI)	*GGATCC*TGACGTTGAACAAACGAATA
lyt-B (HindIII)	*AAGCTT*GATAGCACCTCAGTAAAT
lyt-C(HindIII)	*AAGCTT*CAGTAATCCTTTTTTTTATGC
lyt-D (EcoRI)	*GAATTC*CAACTGTACCGATACCAAT
gra-A (BamHI)	*GGATCC*CAAATAGATATTGCTGTATTC
gra-B (NcoI)	*CCATGG*CCATATCACCCAATATCATT
gra-C (NcoI)	*CCATGG*ACATGCGTTTTGTTACTTAG
gra-D (XhoI)	*CTCGAG*TTAACGCCACCTAAAACAC
sae-A	ATTTGAATGGATAGGC
sae-B	ATTACGTCATAATCCG
sae-C	AAATCGGATTATGACGTAATAAGTGGGTCATCTATTTTTTCACC
sae-D	TAACACTACAAATCGC
tcs7-A (BamHI)	*GGATCC*AAAGGGGATCTAATTATGATA
tcs7-B (HindIII)	*AAGCTT*ATTTTATTCCGCCCTTTTAAA
tcs7-C (HindIII)	*AAGCTT*ATACAAACAAAAAAGTATTGAG
tcs7-D (EcoRI)	*GAATTC*AAAAATACTTCTCTCTAGCAA
arl-A	GTTTCCTTTTTGGAGG
arl-B	TCATGACTGAGACGTC
arl-C	GATTGACGTCTCAGTCATGAGCGTCATTTGTACACC
arl-D	GTGATGAGGTTTAAAC
srr-A (BamHI)	*GGATCC*CCTACAACATTTGTAGCTTT
srr-B (XhoI)	*CTCGAG*AAACTACCAAAACCAGAATAA
srr-C (XhoI)	*CTCGAG*ATAAGTATTTCGTTCGACAT
srr-D (BamHI)	*GGATCC*CATTTTATTCCATAAACCAAC
pho-A	TGGTAGTAAGATACCC
pho-B	AAAGTGGTAACAGCGC
pho-C	ACACGCGCTGTTACCACTTTGGTATGCCTCCCTAAC
pho-D	CACATGGTACAGCTCC
air-A	TTAAAATGTGTGTAATTGCA
air-B (HindIII)	*AAGCTT*CAAATCGCTCCAATTCATTT
air-C (HindIII)	*AAGCTT*AATGAGCTTTTAAATATTTGTC
air-D	GTGTGTTACATCGCTTTTA
vra-A	GTATTACCAGGTGCAG
vra-B	TCAATAGTTCGTATTG
vra-C	AATTCAATACGAACTATTGACGATAAATCACCTCTA
vra-D	ACGTGGCCTTTTGGCG
agr-A	AGCACTGAGTCCAAGG
agr-B	TTTTACACCACTCTCC
agr-C	GTGAGGAGAGTGGTGTAAAAAAGATAATAAAGTCAGTTAACGGC
agr-D	CAGTTATTAGCAGGAT
kdp-A (BamHI)	*GGATCC*CCAATGATATTAGTTAATCCA
kdp-B (NcoI)	*CCATGG*AACCTTCACCTCGATAGC
kdp-C (NcoI)	*CCATGG*CACAATGTCATGAGGACG
kdp-D (EcoRI)	*GAATTC*GTTTTCAATAATTGATTCTCTG
kdp-RN1-A (BamHI)	CGC*GGATCC*CCAATGATATTAGTTAATCCAG
kdp-RN1-B	AACCTTCACCTCGATAGC
kdp-RN1-C	GCTATCGAGGTGAAGGTTTTAAATAAAAAAGATCGCTGCC
kdp-RN1-D (EcoRI)	CCG*GAATTC*AAAGAAAAAGTTGAAAACTGGATG
hss-A	CATTAATAGCGACCTC
hss-B	CTCCCTTATCTTTTTC
hss-C	AAATGAAAAAGATAAGGGAGTCTCTACCTCCTGAAA
hss-D	AGGTGTAGTGTGCATC
nre-A (BamHI)	*GGATCC*CGCAACATTAGTAACCAATAT
nre-B (NcoI)	*CCATGG*GACTTACACCCTAATTCATC
nre-C (NcoI)	*CCATGG*AGTTTGAAATTAATATAATTCAGT
nre-D (XhoI)	*CTCGAG*TTATAAACAGCAAGACTTAGAA
bra-A (BamHI)	*GGATCC*GCTGCAGAATCAGTAATATT
bra-B (HindIII)	*AAGCTT*CTATAATCTTCTTCCTTCAAT
bra-C (HindIII)	*AAGCTT*AAACTTTCAATATTGTAAGCATA
bra-D (EcoRI)	*GAATTC*GTGCCATAACAATCTTAACT

Continued on the following page

Supplementary Table 5. Continued.

Oligonucleotide	Sequence
arlS-A (EcoRI)	*GAATTC*ACATGTTTCCTTTTTGGAGG
arlS-B (SalI)	*GTCGAC*TCATGACTGAGACGTCAATC
arlS-C (SalI)	*GTCGAC*TCATCGTATCACATACCCAACG
arlS-D (BamHI)	*GGATCC*GCCGTCAATTAATGGCTTAG
hpt-E	ATCTACTCAAGTACTGCTTT
hpt-F	TGTTTCAAACGTCTTTGTATA
lyt-E	GAAAAGAAAAATGTAGATTTGA
lyt-F	AATAACATCATTGGCAAATTG
gra-E	AAATGACTTGGATTCAGTGT
gra-F	AAAAAGATAGATGGCATAATG
sae-E	GTTGGTGATTTTAGACTTTTA
sae-F	ATAAGATTGTAGAGCACATAA
tcs7-E	AATTGCCGGAAGATGTTAAA
tcs7-F	TATCTTTGTAGGCTTTATCG
arl-E	AGTGATCTGAAACAATTCC
arl-F	AAGAATAGTAAATAAAACGC
srr-E	TTTGTACAAAGGTAGACTTG
srr-F	GGTATTGAATTAGAAGATGG
pho-E	AGATATTTCCGATATAGCGA
pho-F	CAGGTGCAAACATTAATTATG
air-E	CATATAAAGGATCACCAATAA
air-F	CATAGTTATTCATTATACCAC
vra-E	TGACGAACAAGTGAAATGG
vra-F	CGTTCTATTATTGGGATGTG
agr-E	GGGGATGTTATTAATTATGAA
agr-F	TAGTCATTTATACGAAGGGA
kdp-E	TACTAATTAAACATGATAATGG
kdp-F	GAATTCGTTTTCAATAATTGATTCTCTG
hss-E	CATACATTGTGTCGTTTAAAA
hss-F	AACCAATGATTAAGCTAATAAA
nre-E	TTAAGTTCAGCGTCGGATAT
nre-F	AACTTTACATTATTACGATGAAA
bra-E	TACTTTCTGCTTGTTACTGT
bra-F	ACACAAGCGTATATTCAATC
**Constitutive expression of TCSs**	
ArlS-Rv (XmaI AscI)	*GGCGCGCCCGGG*GATTAAAATATGATTTTAAACG
ArlS-Fw (BamHI)	*GGATCC*TGGCGTTGGGTATGTGATA
ArlR-Rv (BamHI XmaI KpnI)	*GGTACC*AATA*CCCGGG*CAAT*GGATCC*TTTGTCATCGTATCACATAC
ArlR-Fw (SalI XhoI)	*GTCGAC*ATTG*CTCGAG*GTAATATGAGGTGTACAAAT
NreC(R)-Rv (BamHI XmaI KpnI)	*GGTACC*AATA*CCCGGG*CAAT*GGATCC*AATTTCAAACTCTAAAACTCTA
NreC(R)-Fw (SalI XhoI)	*GTCGAC*ATTG*CTCGAG*CATACATTGGGGGAATAAAA
NreB(S)-Rv (XmaI AscI)	*GGCGCGCCCGGG*GTATGTTTCAAATTGGAATGT
NreB(S)-Fw (BamHI)	*GGATCC*AGATGAATTAGGGTGTAAGT
SrrB(S)-Rv (XmaI AscI)	*GGCGCGCCCGGG*CAATTTTATTCTGGTTTTGG
SrrB(S)-Fw (BamHI)	*GGATCC*ATTTGAGGTTAAATCTAATG
SrrA(R)-Rv (BamHI XmaI KpnI)	*GGTACC*AATA*CCCGGG*CAAT*GGATCC*ACTATTTAGCCGGCTCATC
SrrA(R)-Fw (SalI XhoI)	*GTCGAC*ATTG*CTCGAG*TGTGTGGGAGGTATGACC
VraS-Rv (XmaI AscI)	*GGCGCGCCCGGG*CTTTAATCGTCATACGAATC
VraS-Fw (BamHI)	*GGATCC*GAGACGTAGAGGTGATTTAT
VraR-Rv (BamHI XmaI KpnI)	*GGTACC*AATA*CCCGGG*CAAT*GGATCC*GAACTATTGAATTAAATTATGT
VraR-Fw (SalI XhoI)	*GTCGAC*ATTG*CTCGAG*AAATAAGGAGGATTCGTATG
GraR-Rv (BamHI XmaI KpnI)	*GGTACC*AATA*CCCGGG*CAAT*GGATCC*CAAATTATTCATGAGCCATA
GraR-Fw (SalI XhoI)	*GTCGAC*ATTG*CTCGAG*AATGATATTGGGTGATATGG
GraS-Rv (XmaI AscI)	*GGCGCGCCCGGG*CGCATGTTTAAAATGACAAA
GraS-Fw (BamHI)	*GGATCC*TAGGAAAAGGATATATGGCT
HptS-Rv (XmaI AscI)	*GGCGCGCCCGGG*TACCTTAAACATCTACATTC
HptS-Fw (BamHI)	*GGATCC*TTTTTGGAGATGATTCAATG
LytS-Rv (XmaI AscI)	*GGCGCGCCCGGG*TATTTATTCCTCCTCTTGTC
LytS-Fw (BamHI)	*GGATCC*AATTTACTGAGGTGCTATCG
SaeS-Rv (XmaI AscI)	*GGCGCGCCCGGG*ATCGGATTATGACGTAATGT
SaeS-Fw (BamHI)	*GGATCC*ATTTGAAAGGAGCCGATAAT
TCS7S-Rv (XmaI AscI)	*GGCGCGCCCGGG*AAAGATGTCATGCTATTCCT
TCS7S-Fw (BamHI)	*GGATCC*AAAGGGCGGAATAAAATATG
PhoR(S)-Rv (XmaI AscI)	*GGCGCGCCCGGG*TTTTATTCTTTATAATCTTTTAG
PhoR(S)-Fw (BamHI)	*GGATCC*ATTGGAAAGACCTAAAGAAC

Continued on the following page

Supplementary Table 5. Continued.

Oligonucleotide	Sequence
AirS-Rv (XmaI AscI)	*GGCGCGCCCGGG*GCTATTTTATAGGAATTGTG
AirS-Fw (BamHI)	*GGATCC*AAATGAATTGGAGCGATTTG
AgrC(S)-Rv (XmaI AscI)	*GGCGCGCCCGGG*GGCTAGTTGTTAATAATTTC
AgrC(S)-Fw (BamHI)	*GGATCC*TATAAGAGAAAGTGTGATAG
KdpD(S)-Rv (XmaI AscI)	*GGCGCGCCCGGG*CATTATACGTCTCCTTCATT
KdpD(S)-Fw (BamHI)	*GGATCC*TATCGAGGTGAAGGTTATG
HssS-Rv (XmaI AscI)	*GGCGCGCCCGGG*AGATTAAAGTGAATTATTTGG
HssS-Fw (BamHI)	*GGATCC*CAAGGCTATAAGGTGGAGA
BraS-Rv (XmaI AscI)	*GGCGCGCCCGGG*TTTTTATTCATCTGGAAATTG
BraS-Fw (BamHI)	*GGATCC*AGTATAGGGTGAATGCAATG
pSD3.3.1	AAGCTTTTCCCGGGTTTC
WalR-Rv	CCTCTACTCATGTTGTTGGA
**Amino acid substitution**	
ArlR(D52A)-Fw	G**CA**TTAATGTTGCCGTCAATTAATG
ArlR(D52A)-Rv	CATTAATTGACGGCAACATTAA**TG**CTAATATGATTAAATCATAGTA
ArlS(H242A)-Fw	**GCA**GAATTACGAACACCATTACAAATTA
ArlS(H242A)-Rv	TAATTTGTAATGGTGTTCGTAATTC**TGC**TGACGCATCTTCAACAAATTG
**RT-qPCR**	
spa-Fw	GCAAACGGCACTACTGCTGA
spa-RV	CACCAGTTTCTGGTAATGCTTGAG
gyr-Fw	TTATGGTGCTGGGCAAATACA
gyr-Rv	CACCATGTAAACCACCAGATA

Italic, restriction enzyme site included in the oligonucleotide.
Underlined the complementary sequence used to fuse AB PCR products and CD PCR products to generate the mutant alleles.
Bold, nucleotidic changes to generate an amino acid substitution in the corresponding protein.

SUPPLEMENTARY DATA

https://doi.org/10.6084/m9.figshare.5733495.v1

Supplementary Data 1. Quantitative metabolomic results.

SUPPLEMENTARY REFERENCES

1. Valle, J. *et al.* SarA and not sigmaB is essential for biofilm development by *Staphylococcus aureus*. *Mol Microbiol* **48,** 1075–1087 (2003).
2. Ubeda, C., Barry, P., Penadés, J. R. & Novick, R. P. A pathogenicity island replicon in *Staphylococcus aureus* replicates as an unstable plasmid. *Proc Natl Acad Sci U S A* **104,** 14182–14188 (2007).
3. Toledo-Arana, A. *et al. Staphylococcus aureus* develops an alternative, *ica*-independent biofilm in the absence of the *arlRS* two-component system. *J Bacteriol* **187,** 5318–5329 (2005).
4. Novick, R. P. *Molecular biology of the staphylococci*. (1990).
5. Memmi, G., Nair, D. R. & Cheung, A. Role of ArlRS in autolysis in methicillin-sensitive and methicillin-resistant *Staphylococcus aureus* strains. *J. Bacteriol.* **194,** 759–767 (2012).
6. Baba, T. *et al.* Genome and virulence determinants of high virulence community-acquired MRSA. *The Lancet* **359,** 1819–1827 (2002).
7. Dubrac, S., Boneca, I. G., Poupel, O. & Msadek, T. New insights into the WalK/WalR (YycG/YycF) essential signal transduction pathway reveal a major role in controlling cell wall metabolism and biofilm formation in *Staphylococcus aureus*. *J Bacteriol* **189,** 8257–8269 (2007).
8. Arnaud, M., Chastanet, A. & Débarbouillé, M. New vector for efficient allelic replacement in naturally nontransformable, low-GC-content, Gram-positive bacteria. *Appl Environ Microbiol* **70,** 6887–6891 (2004).
9. Dubrac, S. & Msadek, T. Identification of genes controlled by the essential YycG/YycF two-component system of *Staphylococcus aureus*. *J Bacteriol* **186,** 1175–1181 (2004).
10. Charpentier, E. *et al.* Novel cassette-based shuttle vector system for Gram-positive bacteria. *Appl Environ Microbiol* **70,** 6076–6085 (2004).
