# Supplementary material for: Sensory deprivation in Staphylococcus aureus
Source: Nat Commun. 2018 Feb 6;9:523. doi: 10.1038/s41467-018-02949-y (PMC5802764; doi:10.1038/s41467-018-02949-y)
Supplement: Supplementary file 2 — Description of Additional Supplementary Files [file 41467_2018_2949_MOESM2_ESM.pdf]

Description of Additional Supplementary Files

File Name: Supplementary Data 1
Description: The supplementary dataset includes the summary of the phenotypic differences in the metabolome of wild type S. aureus MW2 and its corresponding S. aureus ΔXV mutant derivative.
